# Support‐Intensified Ir─P/O─Mo Cooperative Linkages for Robust Acidic Water Dissociation

**DOI:** 10.1002/advs.202521057

**Published:** 2025-12-05

**Authors:** Jun Mei, Ruipeng Guo, Di Wang, Jing Shang, Jiahao Zhao, Juan Bai, Qianqian Yao, Shixue Dou

**Affiliations:** ^1^ Key Laboratory for Special Functional Materials of Ministry of Education National & Local Joint Engineering Research Center for High‐efficiency Display and Lighting Technology School of Nanoscience and Materials Engineering Henan University Zhengzhou 450046 China; ^2^ School of Materials Science and Engineering Shaanxi University of Science and Technology Xi'an 710021 China; ^3^ School of Chemistry and Physics Centre for Materials Science Queensland University of Technology 2 George Street Brisbane Queensland 4000 Australia; ^4^ Institute of Energy Materials Science University of Shanghai for Science and Technology 516 Jungong Road Shanghai 200093 China; ^5^ Institute for Superconducting and Electronic Materials University of Wollongong Wollongong New South Wales 2522 Australia

**Keywords:** acid, catalyst, interface, molybdenum, phosphorus

## Abstract

The efficient production of hydrogen via acidic water electrolysis is hampered by the sluggish kinetics of the oxygen evolution reaction (OER) and the scarcity of robust bifunctional catalysts. Iridium‐based materials have been recognized as promising active sites; however, the atomic utilization should be maximized, and the stability requires further enhancement. This work introduces a support‐intensified catalyst design that features covalent Ir‐P‐Mo linkages for achieving robust water dissociation. Theoretical calculations reveal that the Ir─P─Mo bond enhances hydrogen evolution reaction (HER) activity by optimizing hydrogen adsorption, while the Ir─O─Mo bond is more favorable for OER. Guided by this principle, a catalyst with coexisting Ir‐O‐Mo and Ir‐P‐Mo linkages is rationally synthesized, which exhibits exceptional bifunctional performance in acid solution, including low overpotentials of 33 mV for HER and 249 mV for OER at 10 mA cm^−2^. When configured in a symmetrical two‐electrode electrolyzer, it requires only 1.501 V to reach 10 mA cm^−2^ and demonstrates remarkable stability for 250 h with minimal voltage degradation. This work verifies the critical role of interfacial bond engineering in developing efficient and durable iridium‐based electrocatalysts for practical acidic water splitting.

## Graphical abstract

An electrocatalyst with the coexisting Ir‐O‐Mo and Ir‐P‐Mo linkages is rationally synthesized for achieving exceptional bifunctional performance in acid solution.

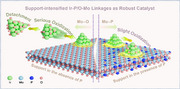

## 1 Introduction

Currently, an efficient transition toward sustainable energy sources is definitely a major challenge, in which the replacement of fossil fuels with clean energy is a strategic solution, such as hydrogen production.^[^ ^1^, ^2^ ^]^ A promising pathway for producing hydrogen is electrolytic water dissociation that is generally powered by renewable electricity such as solar or wind.^[^ ^3^ ^]^ In this process, water is disassociated into hydrogen and oxygen in the presence of efficient electrocatalysts, and the overall efficiency is critically dependent on the kinetics of the two half‐reactions: hydrogen evolution reaction (HER) and oxygen evolution reaction (OER). While the two‐electron HER is readily achieved in acidic media, the four‐electron OER is relatively complex, which suffers from intrinsically high overpotentials.^[^ ^4^, ^5^, ^6^ ^]^ Moreover, the harsh acidic environment imposes strict constraints on the electrocatalysts that can be employed, especially for OER on the anode.^[^ ^7^ ^]^ Thus, the request for electrocatalysts that can efficiently and sustainably catalyze water dissociation in acidic media is a fundamental prerequisite for large‐scale production of green hydrogen.

Iridium (Ir) based electrocatalysts have been recognized as potential materials for acidic OER, which is largely due to a compromise between catalytic activity and structural stability.^[^ ^8^, ^9^ ^]^ However, as one of the noble metals, the natural scarcity of iridium presents a great barrier for practical applications.^[^ ^10^, ^11^ ^]^ To maintain high performance at minimal iridium loading without compromising the foundational requirement of long‐term durability under acidic conditions, research efforts are dedicated to maximizing the utilization efficiency of iridium atoms, either by creating nanosized structures, designing mixed metal oxides, or developing support‐type catalysts.^[^ ^12^, ^13^, ^14^ ^]^ Particularly, the engineering of iridium centers on oxide supports to form support‐type catalysts has been proven as a promising solution, in which the electrochemically active surface is maximized, accompanied by the induced strong metal‐support interactions (SMSI), which facilitates modulating the electronic structure of iridium sites and enhancing their intrinsic catalytic activity.^[^ ^15^, ^16^ ^]^ On the other hand, the presence of oxide supports promotes the dispersion of iridium sites for improving mass activity, and provides remarkable corrosion resistance for retaining structural integration in acidic media.^[^ ^17^, ^18^, ^19^ ^]^ For example, anchoring iridium sites onto metal oxide support could intrigue interfacial Ir─M or Ir─O─M bonding is evidenced to considerably promote catalytic activity for acidic water dissociation.^[^ ^20^ ^]^ Unfortunately, some major drawbacks remain, such as the physical or weakly chemical interaction between iridium sites and the support, and the structural stability under continuous operation in acidic solutions.^[^ ^21^, ^22^ ^]^ Therefore, an ideal iridium‐based supported catalyst under acidic conditions should achieve high dispersion of active iridium sites to increase atomic utilization ratios, forge strong and durable chemical linkages to accommodate undesired degradation, and induce electronic optimization on active centers for acidic water dissociation.^[^ ^23^, ^24^, ^25^ ^]^

To address these challenges, a strategic design of support‐intensified Ir‐P‐Mo linkages is proposed in this work, in which a covalent phosphorous bridge is formed between the iridium active site and the molybdenum oxide support. Coupled with the existing Ir‐O‐Mo linkages, the accelerated electron transfer process is expected to optimally weaken the binding strength of oxygenated intermediates and thereby enhance the intrinsic activity for the rate‐limiting water‐dissociation step.^[^ ^26^ ^]^ Besides, the interfacial phosphorus bridges in Ir‐P‐Mo linkages are favorable for achieving a cooperative bifunctional reaction mechanism that further lowers kinetic barriers. In contrast to the previous individual Ir─P and Ir─O bonds, the P/O unit creates an asymmetric and polarized bridge between the dissimilar Ir and Mo metals, which is much favorable for achieving synergistic catalysis. Considering the significant effects from the support, guided by theoretical screening results, two types of molybdenum oxide supports with different surface chemistry states are rationally synthesized for anchoring iridium sites. By comparison, it is concluded that the co‐existence of Ir‐O‐Mo and Ir‐P‐Mo linkages could enhance the OER stability using Ir‐O‐Mo and the HER activity using Ir‐P‐Mo, which endows the PMoO_EtOH catalyst as an efficient bifunctional material for overall water splitting. As a result, the optimal catalyst shows a low overpotential of 33 mV for generating hydrogen under acidic conditions, a low operation voltage of only 1.501 V for achieving 10 mA cm^−2^ in the acidic solution, and a voltage retention of 96.34% over 250 h. This innovative approach provides a promising solution for retaining activity and structural integration at an economic level for support‐type catalysts.

## 2 Results and Discussion

**Figure 1:**
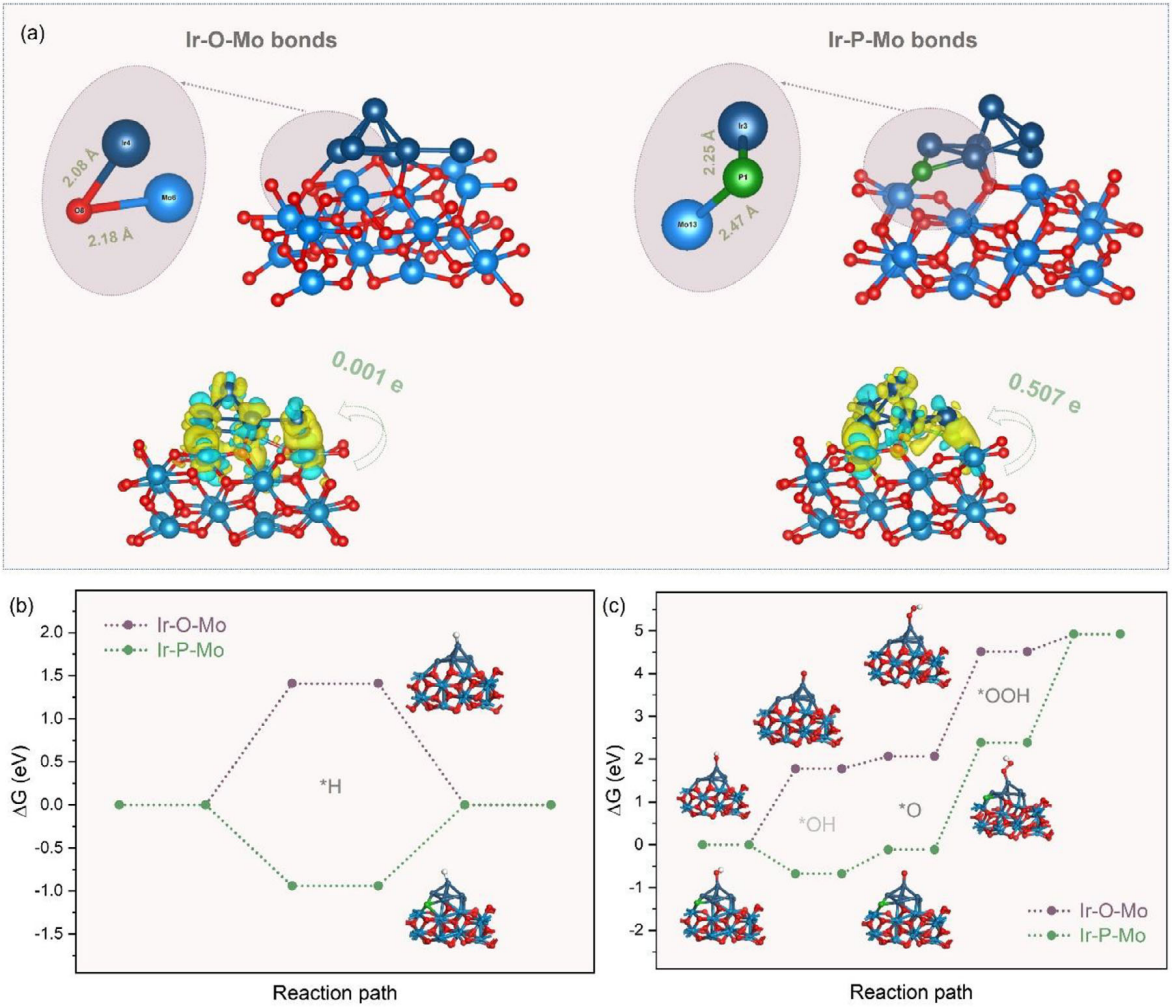
Theoretical analysis on functional roles of Ir‐O‐Mo and Ir─P─Mo bonds for OER and HER. a) Theoretical illustration of Ir‐O‐Mo and Ir‐P‐Mo models and the corresponding differential charge density patterns. b, c) Intermediate adsorption energies for (b) HER and (c) OER.

To well design the target catalyst for OER and HER, the functional roles of Ir─O─Mo and Ir─P─Mo bonds were theoretically studied using density functional theory (DFT). As compared in **Figure** **1**a, the bond length of Ir‐O (2.08 Å) is much shorter than that of Ir‐P (2.25 Å) in the presence of the Mo site. Based on the differential charge density patterns, the Ir site only receives a small electron number of 0.001 in the Ir‐O‐Mo model, which is significantly increased to 0.507 in the Ir‐P‐Mo model. In order to compare the different influences of Ir‐O‐Mo and Ir─P─Mo bonds on catalytic activities, the intermediate adsorption energies during HER and OER are compared. For HER (Figure 1b), the Gibbs free energy (ΔG) for the reaction intermediate (H^*^) in the Ir‐O‐Mo model is 1.41 eV, suggesting an unfavorable adsorption behavior; however, this was changed to be −0.94 eV after the incorporation of phosphorus in the Ir‐P‐Mo model, implying a favorable adsorption for HER. In contrast, for OER (Figure 1c), the Ir‐P‐Mo model exhibits a larger energy barrier of 2.62 eV than that in the Ir‐O‐Mo model (2.38 eV), accompanied by the shift on the rate‐determination step (RDS) from ^*^OOH → O_2_ to ^*^OH → ^*^OOH, which clearly indicates the Ir─O─Mo bonds are more energetic for OER. Hence, it can be inferred that the co‐existence of Ir─O─Mo and Ir─P─Mo bonds is ideal for catalytic OER and HER.

**Figure 2:**
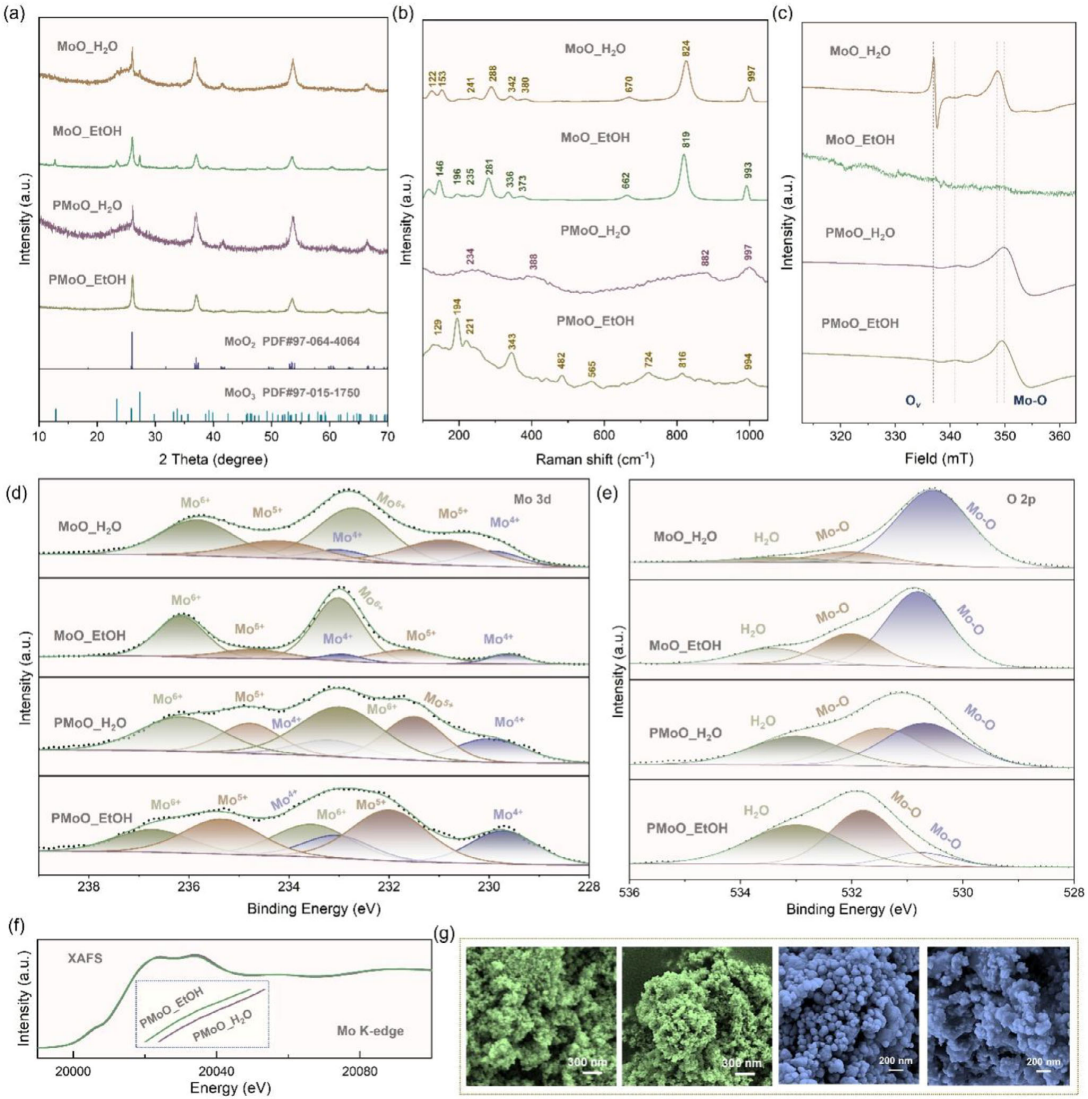
Structural characterizations of the MoO_H_2_O and MoO_EtOH precursors, and the PMoO_H_2_O and PMoO_EtOH supports. a) XRD patterns of the precursors and the supports. b) Raman spectra of the precursors and the supports. c) Electron paramagnetic resonance (EPR) plots of the precursors and the supports. d, e) High‐resolution XPS spectra at (d) Mo 3d and (e) O 2p the precursors and the supports. f) Fourier‐transform edge extended X‐ray absorption fine structure (EXAFS) spectra of the PMoO_H_2_O and PMoO_EtOH supports. g) SEM images of the precursors and the supports.

To prepare the electrocatalyst with a mixed Ir─O─Mo and Ir─P─Mo bonds as predicted by theoretical results, the Mo‐(P)‐O support was first synthesized by a controllable surface regulation strategy. Specifically, a polymer‐assisted molecular self‐assembly approach was used in the presence of different solvent systems (e.g., water or water‐ethanol), and then the obtained precursor (MoO_H_2_O or MoO_EtOH) was annealed and phosphorization to produce the required support (PMoO_H_2_O or PMoO_EtOH). By adjusting the solvent systems, the arrangement of Mo atoms could be modulated, which causes the changes in surface chemistry. X‐ray diffraction (XRD) was used to examine the phase transition process before and after the incorporation of phosphorus, as illustrated in **Figure** **2**a, in which the dominant phases in the MoO_H_2_O and MoO_EtOH precursors consist of monoclinic MoO_2_ (PDF#97‐064‐4064) and orthorhombic MoO_3_ (PDF#97‐015‐1750). After phosphorization, dual‐phase precursors are transformed into MoO_2_ to produce the PMoO_H_2_O and PMoO_EtOH supports, which are largely due to the strong reduction reactions from high‐valence Mo (VI) to low‐valence Mo (IV) in the presence of phosphorus. In spite of similar phases, the specific states are distinctly different. For example, the crystalline state for the PMoO_H_2_O support is relatively weaker than that for the PMoO_EtOH support. This can also be confirmed by Raman spectra (Figure 2b).

Except for the obvious phase transition, the incorporation of phosphorus was simultaneously achieved during phosphorization, as verified by electron paramagnetic resonance (EPR) plots (Figure 2c), the MoO_H_2_O precursor shows more obvious EPR signals associated with oxygen and molybdenum vacancies than that for the MoO_EtOH precursor, however, the oxygen vacancies in both PMoO_H_2_O and PMoO_EtOH supports become considerably weak, which is largely due to the successful insertion of phosphorus into Mo‐O frameworks. Moreover, X‐ray photoelectron spectroscopy (XPS) was applied to evidence surface chemistry before and after phosphorization. As compared in the XPS survey spectra, additional phosphorus signals are clearly observed in the PMoO_H_2_O (Figure S1, Supporting Information), and PMoO_EtOH (Figure S2, Supporting Information) supports in comparison with the corresponding MoO_H_2_O and MoO_EtOH precursors, indicating the successful insertion of phosphorus atoms into MoO_2_ frameworks. As shown in the Mo 3d XPS spectra (Figure 2d), the MoO_EtOH precursor possesses less low‐valence Mo (IV) and Mo (V), while the MoO_H_2_O precursor possesses less high‐valence Mo (VI), which is in good agreement with EPR results, in which more oxygen vacancies were found in the MoO_H_2_O precursor for inducing more low‐valence Mo. After phosphorizing into the PMoO_H_2_O and PMoO_EtOH supports, the ratios of high‐valence Mo decrease, but the specific case is distinctly different. Compared to the PMoO_H_2_O support, the PMoO_EtOH support delivers a higher percentage of low‐valence Mo(IV) and Mo (V), amounting to over 70% (Figure S3, Supporting Information), which indicates the stronger reduction for the MoO_EtOH precursor. Further comparison on the O 1s XPS spectra (Figure 2e) demonstrates that the lattice oxygen located at ≈530 eV is significantly reduced from the original 60–80% in the precursors into 10–40% after phosphorization, and the PMoO_EtOH support holds more obvious Mo‐O binding states that are highly related to vacancies appearing at ≈532.5 eV. In terms of P states, the Mo─P─(O) bonding can be confirmed in the PMoO_H_2_O and PMoO_EtOH supports, and the XPS signal in the PMoO_EtOH support is more evident (Figure S4, Supporting Information). Also, Fourier transform infrared spectroscopy (FT–IR) spectra of the PMoO_H_2_O and PMoO_EtOH support demonstrates different vibration modes of Mo‐O that might be caused by the local environment varieties in the presence of phosphorus (Figure S5, Supporting Information). Besides, X‐ray absorption near‐edge spectroscopy (XANES) plots (Figure 2f) show a more negative pre‐edge state for the PMoO_EtOH support compared to the PMoO_H_2_O support, implying the decreased average valence state contributed by the strong Mo‐P interaction.

Finally, the SEM technique was applied to check the morphological changes before and after annealing or phosphorization. It is clearly observed in Figure 2g that the MoO_H_2_O precursor before annealing displays a rough flower‐like morphology (Figure S6, Supporting Information) while the MoO_EtOH precursor exhibits a smooth sphere‐like morphology (Figure S7, Supporting Information), which were partially or completely destroyed into disordered structures due to the severe phosphorization reactions between Mo and P. Hence, these characterization results indicate that the introduced phosphorization reaction can induce the disordered morphology, incorporation of phosphorus, and the reduction of Mo, accompanied by the formation of Mo─P bonds, which will pose crucial influences on the subsequent loading of metallic sites.

**Figure 3:**
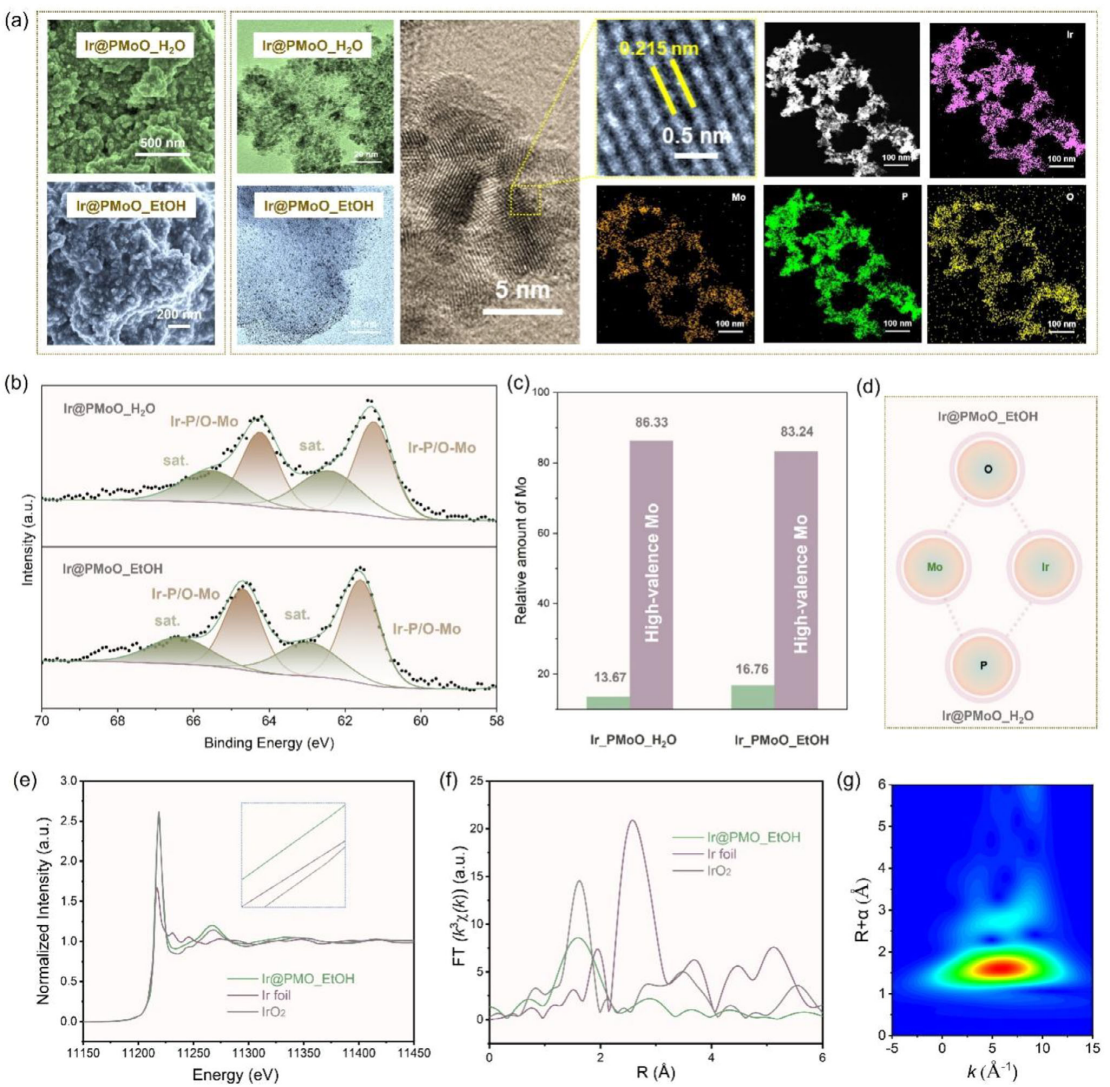
Structural characterizations of the Ir@PMoO_H_2_O and Ir@PMoO_EtOH catalysts. a) SEM images, TEM images, HRTEM images, and high‐angle annular dark‐field TEM (HAADF‐TEM) image and element mapping patterns in the Ir@PMoO_H_2_O and/or Ir@PMoO_EtOH catalysts. b) High‐resolution XPS spectra of Ir 4f in the Ir@PMoO_H_2_O and Ir@PMoO_EtOH catalysts. c) XPS ratio comparison on Mo in the Ir@PMoO_H_2_O and Ir@PMoO_EtOH catalysts. d) Schematic illustration of the Ir‐O/P‐Mo linkages in Ir@PMoO_H_2_O and Ir@PMoO_EtOH catalysts. e) XANES spectra at Ir L‐edge, and f) Fourier‐transform edge extended X‐ray absorption fine structure (EXAFS) spectra of the Ir@PMoO_EtOH catalysts, IrO_2,_ and Ir foil. g) Wavelet transforms contour plots of the Ir@PMoO_EtOH catalysts.

After loading with noble‐metal Ir sites onto the PMoO_H_2_O and PMoO_EtOH supports by a thermal reduction approach, the Ir@PMoO_H_2_O and Ir@PMoO_EtOH catalysts were obtained with a similar Ir/Mo molar ratio of 1:4.3, as confirmed by the inductively coupled plasma optical emission spectrometer (ICP‐OES) measurement (Table S1, Supporting Information). During the synthesis, Ir^3+^ ions from the chlorinated precursor were adsorbed onto the surface of the PMoO_H_2_O or PMoO_EtOH support, which was facilitated by electrostatic interactions and the reactive surface of the support. Then, the heating operation promoted the thermal decomposition of the Ir‐containing salts, and the hydrogen gas acted as a reducing agent to convert the Ir^3+^ ions into metallic iridium (Ir^0^), which was nucleated and grown into larger and stable Ir nanoparticles anchored onto the support.^[^ ^27^, ^28^, ^29^ ^]^ As shown in **Figure** **3**a, the morphological and structural characterizations were conducted using SEM and TEM. First, SEM images and the corresponding element mapping patterns of the Ir@PMoO_H_2_O (Figure S8, Supporting Information) and Ir@PMoO_EtOH (Figure S9, Supporting Information) catalysts show the uniform distribution of Ir particles on the support. Notably, a phosphorus doping level of 4.7 and 2.78 wt% was identified for the PMoO_H_2_O (Figure S10, Supporting Information) and PMoO_EtOH (Figure S11, Supporting Information) supports, respectively. Then, TEM images offer more details, such as the particle size of  2—3 nm of Ir particles (Figure S12, Supporting Information), the crystal lattice of 0.215 nm attributed to metallic Ir, and the homogeneous element distribution of Ir, Mo, P, and O in the Ir@PMoO_H_2_O and Ir@PMoO_EtOH catalysts (Figure S13, Supporting Information). Subsequently, XPS spectra of Ir 4f (Figure 3b) show the formation of Ir─P/O─Mo bonding in these two catalysts; however, the binding energy for the Ir@PMoO_EtOH catalyst (61.59 eV) is slightly higher than that for the Ir@PMoO_H_2_O catalyst (61.24 eV).^[^ ^30^, ^31^ ^]^ Furthermore, in the Mo 3d XPS spectra (Figure S14, Supporting Information), a higher energy is identified in the Ir@PMoO_EtOH catalyst (231.92 eV) compared to the Ir@PMoO_H_2_O catalyst (231.33 eV), and a relatively high ratio of low‐valence molybdenum (Figure 3c). In the O 2p XPS spectra (Figure S15, Supporting Information), the signal attributed to the lattice oxygen appeared at a higher binding energy in the Ir@PMoO_EtOH catalyst (530.68 eV) than that in the Ir@PMoO_H_2_O catalyst (530.51 eV), which is largely due to the oxygen in the Ir‐O‐Mo bridge that is subjected to the combined electron‐withdrawing effect of two highly charged cations loses more electron density than oxygen bonded only to molybdenum (Mo‐O). For the P 2p XPS spectra (Figure S16, Supporting Information), the typical peaks in the Ir@PMoO_H_2_O and Ir@PMoO_EtOH catalysts shift to lower energies after being loaded with metallic Ir sites, indicating the restriction of O─P─Mo bonds and the formation of Ir─P─Mo bonds. Considering a much higher electronegativity of oxygen than phosphorus, the Ir─O bond causes the partial Ir atom to lose electrons, resulting in a higher binding energy of Ir in Ir‐O‐Mo in comparison to Ir‐P‐Mo. Also, molybdenum in the Ir‐O‐Mo structure is surrounded by the highly electronegative oxygen ligands, and its electron cloud density is further reduced by the positively charged iridium atoms, resulting in a higher binding energy than that in the Ir‐P‐Mo structure. Hence, the Ir@PMoO_EtOH catalyst possesses more Mo─O─Ir bonding while the Ir@PMoO_H_2_O catalyst possesses more Mo─P─Ir bonding (Figure 3d).

X‐ray absorption near‐edge spectroscopy (XANES) was applied to examine the local chemistry environment of the Ir@PMoO_EtOH catalysts in comparison to Ir foil and IrO_2_. Figure 3e shows the Ir L3‐edge XANES spectra, in which the Ir@PMoO_EtOH catalyst exhibits a more negative pre‐edge line than that for Ir foil and IrO_2_, indicating the strong Ir‐Mo interaction. As compared in the extended X‐ray absorption fine structure (EXAFS) spectra (Figure 3f), except for Ir─Ir bonds, Ir─P─Mo and Ir─O─Mo bonds are identified in the Ir@PMoO_EtOH catalyst. Based on the EXAFS refinement curves (Figure S17, Supporting Information) and the wavelet transforms contour map (Figure 3g), the Ir@PMoO_EtOH catalyst shows a small Ir‐Ir coordination number of 3.7, which is significantly lower than that of Ir foil (Table S2, Supporting Information), suggesting that the distribution of small Ir clusters is highly dispersed on the support. Also, the high Ir‐O‐Mo coordination number shows the presence of abundant oxidized surface, and the detection of the Ir‐P‐Mo shell indicates a strong covalent interaction between the Ir sites and the P‐doped oxide support.

**Figure 4:**
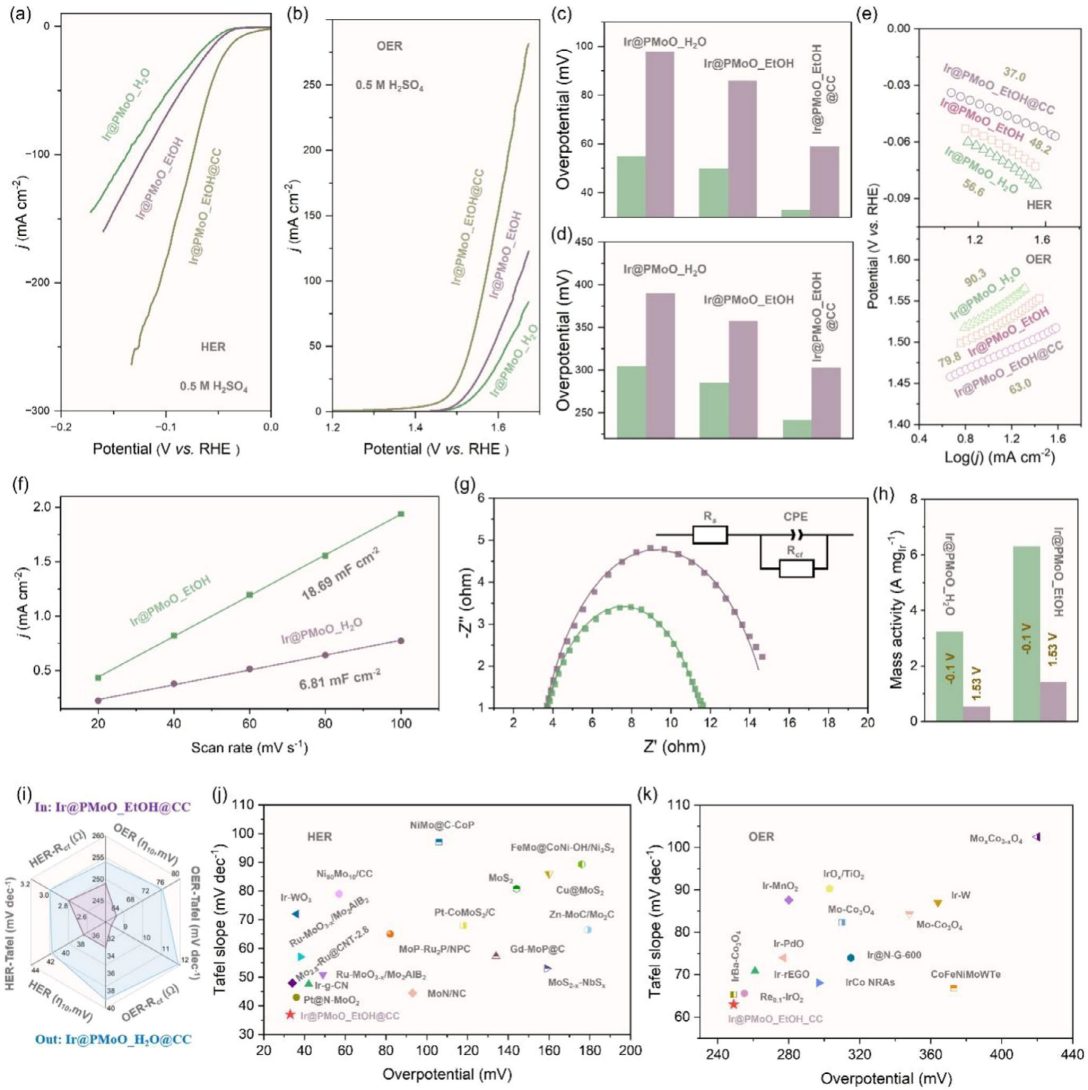
Electrocatalytic characterizations of the Ir@PMoO_H_2_O and Ir@PMoO_EtOH catalysts for acidic HER and OER in 0.5 m H_2_SO_4_ solution. a, b) iR‐corrected linear sweep voltammetry (LSV) plots for acidic (a) HER and (b) OER. c, d) Overpotential comparison of the Ir@PMoO_H_2_O and Ir@PMoO_EtOH catalysts at 10 mA cm^−2^ for (c) HER and (d) OER. e) Tafel slope of Ir@PMoO_H_2_O, Ir@PMoO_EtOH, and Ir@PMoO_EtOH@CC catalysts for HER and OER. f) Comparison of the C*_dl_* values as an indicator for ECSA. g) EIS spectra of Ir@PMoO_H_2_O and Ir@PMoO_EtOH catalysts and the corresponding equivalent circuit diagram. h) Mass activity of Ir@PMoO_H_2_O and Ir@PMoO_EtOH catalysts for HER (at −0.1 V) and OER (at 1.53 V). i) Comparison of several crucial parameters for OER and HER of two catalysts. j, k) Comparison of catalytic activities of Ir@PMoO_H_2_O and Ir@PMoO_EtOH catalysts with these previously reported catalysts in acidic solutions.

Electrocatalytic activities of the Ir@PMoO_H_2_O and Ir@PMoO_EtOH catalysts for acidic water dissociation were assessed in the acidic solution (0.5 m H_2_SO_4_). As compared in **Figure** **4**a, the overpotentials of the Ir@PMoO_H_2_O and Ir@PMoO_EtOH catalysts are 55 and 50 mV, respectively, to achieve the current density of 10 mA cm^−2^ for HER. Similarly, for acidic OER (Figure 4b), the Ir@PMoO_H_2_O catalyst shows a higher overpotential (305 mV) than that for the Ir@PMoO_EtOH catalyst (285  mV). Moreover, the merits of the Ir@PMoO_EtOH catalyst are obvious when the current density increased to 50 mA cm^−2^, in which only 86 mV for HER (Figure 4c) and 358 mV for OER are required (Figure 4d), outperforming the Ir@PMoO_H_2_O catalyst (98 mV for HER and 390 mV for OER). If the Ir@PMoO_EtOH catalyst was loaded onto carbon cloth (CC) substrate to produce the Ir@PMoO_EtOH@CC catalyst, the density at a specific potential is significantly enhanced, and the overpotentials are greatly reduced to only 33 mV for HER and 249 mV for OER at the density of 10 mA cm^−2^ under acidic conditions. Even at a high density of 50 mA cm^−2^, the overpotentials of the Ir@PMoO_EtOH@CC catalyst for HER and OER are 59 and 303 mV, respectively, indicating great potential for practical applications. Then, Tafel slopes were calculated to evaluate reaction kinetics, as demonstrated in Figure 4e, the Ir@PMoO_EtOH catalyst presents the lower Tafel slopes of 48.2 mV dec^−1^ for HER and 79.8 mV dec^−1^ for OER, which are superior to the catalyst with a HER slope of 56.6 mV dec^−1^ and an OER slope of 90.3 mV dec^−1^ in 0.5 m H_2_SO_4_ solution. If CC was used, the slopes are further reduced into 63.0 for OER and 37.0 for HER, suggesting the much favorable catalytic kinetics of the Ir@PMoO_EtOH catalyst for both HER and OER.

Electrochemically active surface area (ECSA) values of the Ir@PMoO_H_2_O (Figure S18, Supporting Information) and Ir@PMoO_EtOH (Figure S19, Supporting Information) catalysts were estimated by the double layer capacitance (C*_dl_*) indicator that was obtained based on CV curves within non‐faradaic potential region at different scan rates from 20 to 100 mV s^−1^. As presented in Figure 4f, the calculated C*_dl_* values of the Ir@PMoO_EtOH catalyst are 18.69 mF cm^−2^ in 0.5 m H_2_SO_4_ solution, which is much larger than that for the Ir@PMoO_H_2_O catalyst. Also, the calculated turnover frequency (TOF) values of the Ir@PMoO_EtOH catalyst are 0.46 S^−1^ for OER (Figure S20, Supporting Information) and 0.97 S^−1^ for HER (Figure S21, Supporting Information), respectively, at a current density of 10 mA cm^−2^. Besides, charge transfer resistance (R*_ct_*) was fitted by electrochemical impedance spectroscopy (EIS) plots, in which the Ir@PMoO_EtOH catalyst delivers the R*_ct_* values of only 8.4 and 2.8 ohms for acidic OER (Figure 4g) and HER (Figure S22, Supporting Information), respectively, outperforming the Ir@PMoO_H_2_O catalyst with the values of 11.9 ohms for OER and 3.0 ohms for HER. If only iridium sites are calculated, the mass activities of the Ir@PMoO_EtOH catalyst are 6.31 and 1.43 A mg_Ir_^−1^ for HER and OER, respectively, which are considerably higher than that for the Ir@PMoO_H_2_O catalyst (3.24 A mg_Ir_^−1^ for HER and 0.55 A mg_Ir_^−1^ for OER) (Figure 4h).

To summarize, compared to the Ir@PMoO_H_2_O catalyst with higher overpotentials and Tafel slopes, the Ir@PMoO_EtOH@CC catalyst exhibits better activity for both OER and HER under acidic conditions (Figure 4i). Compared with these previously reported electrocatalysts, the obtained Ir@PMoO_EtOH@CC catalyst is advantageous to many noble and non‐noble metal‐based catalysts for acidic HER (Figure 4j; Table S3, Supporting Information), such as Ru‐MoO_3‐_ *_x_*/Mo_2_AlB_2_,^[^ ^32^ ^]^ and Pt@N‐MoO_2_,^[^ ^33^ ^]^ and acidic OER (Figure 4k; Table S4, Supporting Information), such as CoFeNiMoWTe^[^ ^34^ ^]^ and Ir‐rEGO.^[^ ^35^ ^]^

**Figure 5:**
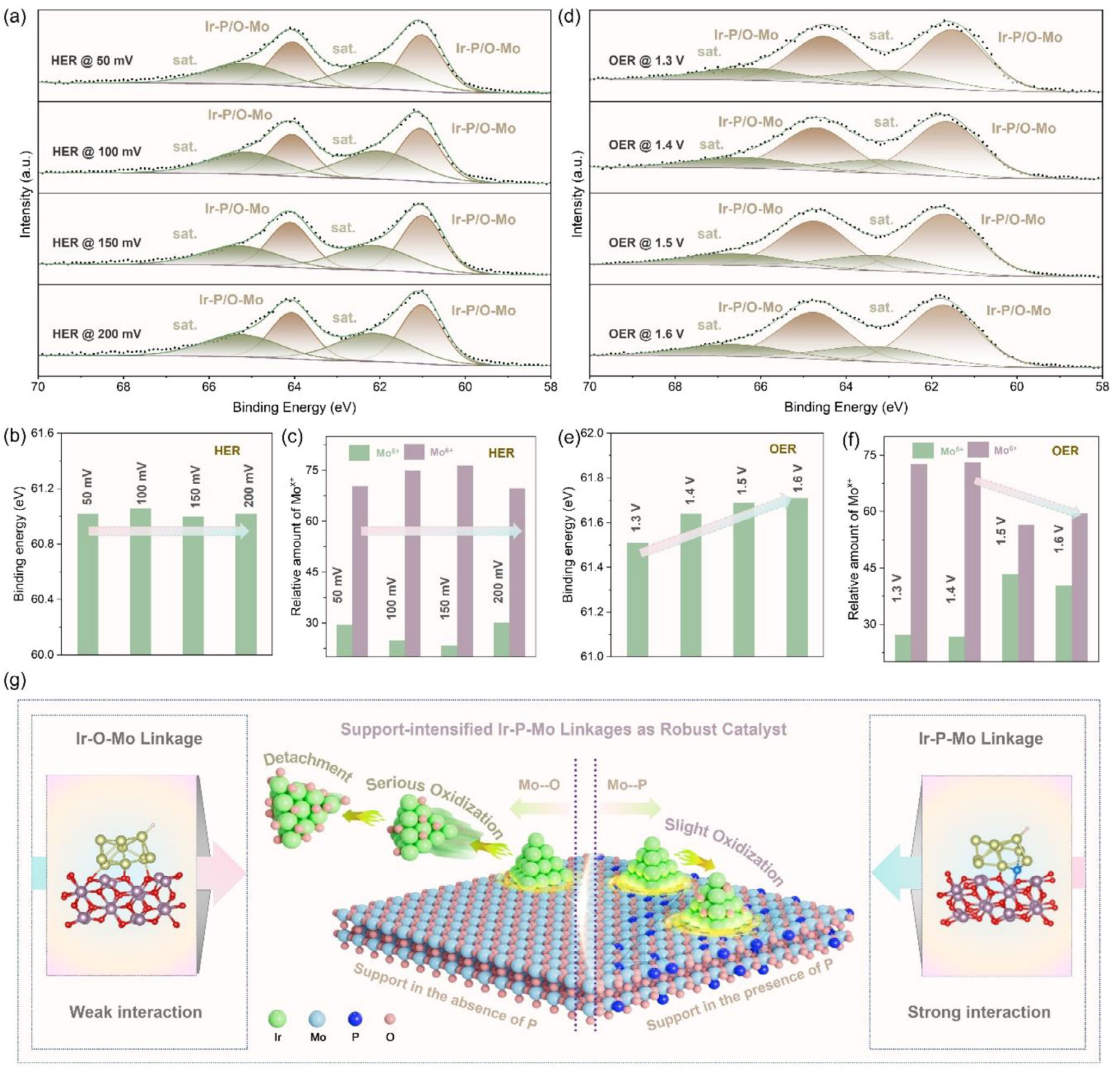
Electrocatalytic mechanism of the resultant Ir@PMoO_EtOH catalyst toward acidic HER and OER. a) Potential‐dependent Ir 4f XPS spectra of the Ir@PMoO_EtOH catalyst for acidic HER, and b, c) Comparison of the ratios of Ir and Mo at differe HER potentials. d) Potential‐dependent Ir 4f XPS spectra of the Ir@PMoO_EtOH catalyst for acidic OER, and e, f) Comparison of the ratios of Ir and Mo at different OER potentials. g) Schematic illustration of the activity enhancement mechanism using support‐intensified Ir‐P/O‐Mo linkages for robust acidic electrocatalysis.

To uncover the activity enhancement mechanism of the resultant Ir@PMoO_EtOH catalyst, the XPS technique was applied to track the variations in surface states during HER and OER under acidic conditions. **Figure** **5**a compares Ir 4f XPS spectra of the Ir@PMoO_EtOH catalyst at a potential range between 50 and 200 mV for acidic HER, in which the binding energy of Ir‐P/O‐Mo remains stable at around 61 eV (Figure 5b) and the chemical environment of Mo is relatively steady (Figures 5c; S23, Supporting Information), accompanied by an obvious reduced P 2p signal. When operated for OER in the acidic solution (Figure 5d), the binding energy for Ir 4f shifts to a higher position as the potential increases (Figure 5e). In contrast, the Mo signal appears at a much lower position when the potential increases to 1.6 V (Figures 5f; S25, Supporting Information), and the typical peak for P 2p becomes wider with a reduced intensity (Figure S26, Supporting Information).

As illustrated in Figure 5g, due to the presence of phosphorus species in the support, the Ir‐P/O‐Mo linkages were rationally built after loading of iridium sites on the PMoO_EtOH support, which is crucial for supporting the catalytic activity in acidic solutions. Specifically, the primary advantage of the Ir‐O‐Mo linkage lies in its exceptional stability under the oxidizing conditions of OER. The Ir─O bond is highly robust, and the Mo‐O support is known to be corrosion‐resistant in acid, which results in the formation of a stable interface that prevents the dissolution of iridium sites. Furthermore, the strong electronic interaction through the oxygen bridge can modulate the electronic structure of the iridium sites, which can optimize the adsorption strength of oxygen intermediates and enhance intrinsic OER activity. However, a significant drawback of this oxide‐based linkage is its inferior performance for HER, in which the oxide support with poor electrical conductivity can impede electron transfer to the iridium sites to induce high overpotentials. Also, as operation time increases, the deep oxidation of iridium sites occurs, thus leading to the detachment of iridium sites from the oxide support. In contrast, the Ir‐P‐Mo linkage introduces a different interface, in which the electron transfer between iridium and the support is greatly enhanced for boosting HER activity and lowering the overpotential. The drawback, however, is the relative instability of the P─Mo bond and the Ir‐P linkage under the high potentials of OER, which is largely due to the possible oxidation to soluble phosphate species over long‐term operation. Therefore, while the Ir‐P‐Mo linkage is favorable for HER, it might compromise the durability for OER. Hence, the co‐existence of Ir‐O‐Mo and Ir‐P‐Mo linkages could enhance the OER stability using Ir‐O‐Mo and the HER activity using Ir‐P‐Mo, which endows the PMoO_EtOH catalyst as an efficient bifunctional material for overall water splitting.

**Figure 6:**
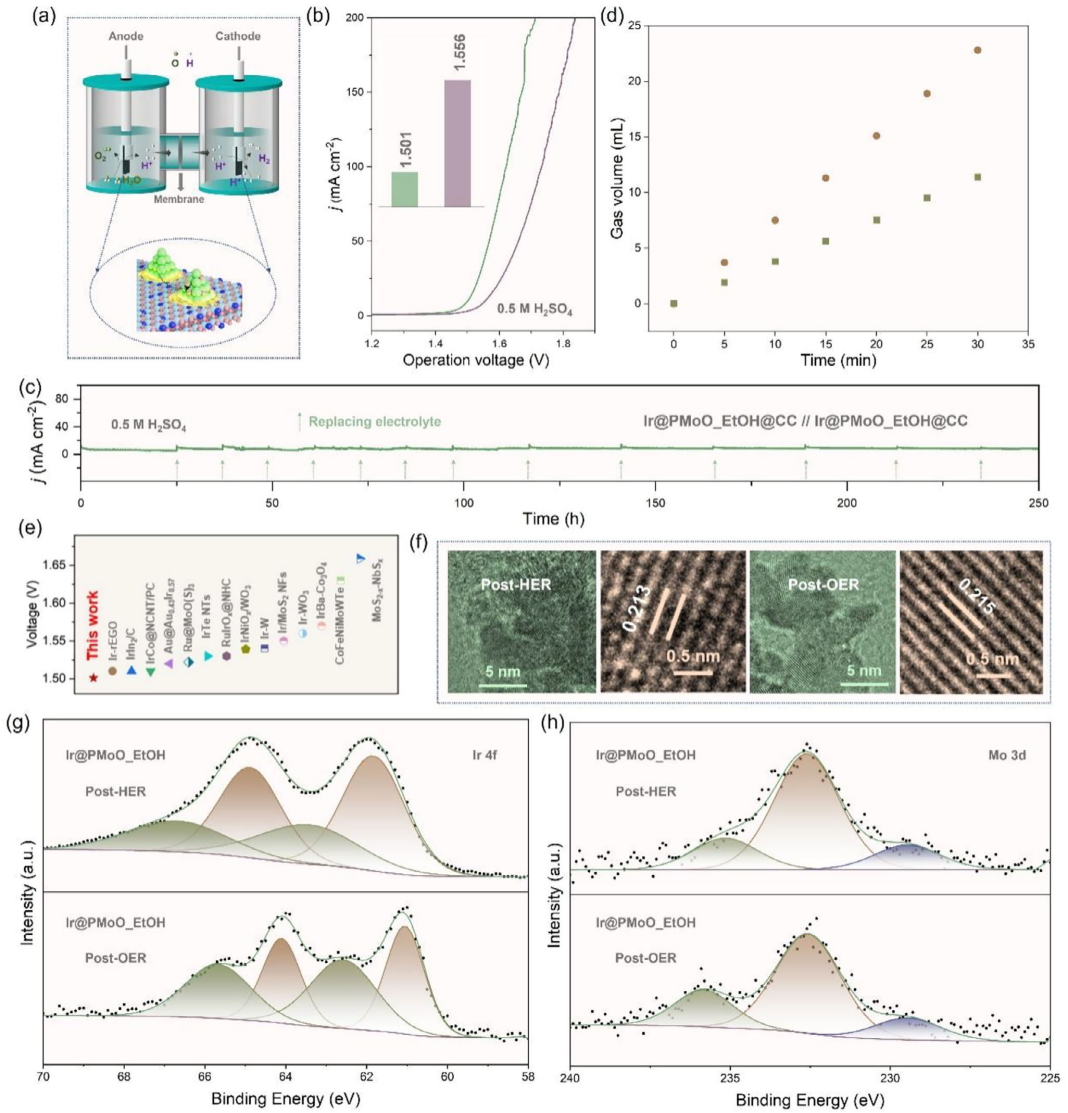
Water dissociation performance of two‐electrode symmetrical cells based on the resultant Ir@PMoO_EtOH@CC catalyst in the acidic solution. a) Schematic illustration of a two‐electrode system for acidic water dissociation using the resultant Ir@PMoO_EtOH@CC catalyst. b) LSV plots and comparison on operation voltages at 10 mA cm^−2^ before and after stability test. c) Chronoamperometric measurement for continuous 250 h in 0.5 m H_2_SO_4_ solutions. d) Generated oxygen and hydrogen gas at different intervals. e) Comparison of the operation voltage of the Ir@PMoO_EtOH@CC based cells with these previously reported two‐electrode systems. f) Low‐ and high‐resolution TEM image and g, h) high‐resolution XPS spectra of Ir 4f and Mo 3d for the Ir@PMoO_EtOH@CC catalyst after long‐life stability measurements.

To evidence the practicability of the resultant Ir@PMoO_EtOH@CC catalyst, a two‐electrode symmetrical H‐type cell was assembled for overall water dissociation under acidic conditions (**Figure** **6**a). As displayed in Figure 6b, to generate the desired density of 10 mA cm^−2^, the required operation voltage is only 1.501 V, and more importantly, after continuous operation up to 250 h in 0.5 m H_2_SO_4_ solution, the voltage increases by only 3.66% (Figure 6c). By measuring the generated gas volume at a fixed interval, Faraday efficiency was also estimated, in which the gas ratio of hydrogen and oxygen is close to 2:1 (Figure 6d). In comparison to these previously reported two‐electrode systems presented in Figure 6e, the operation voltage of the Ir@PMoO_EtOH@CC‐based symmetrical cell is lower than that of Ir‐containing systems (Table S5, Supporting Information), such as IrIn_2_/C//IrIn_2_/C (1.51 V).^[^ ^36^ ^]^

In order to verify the structural stability of the Ir@PMoO_EtOH@CC catalyst, TEM and XPS techniques were employed to examine morphological and structural characteristics after long‐term stability tests in an acid solution. As shown in Figures S27 and S28 (Supporting Information), the active iridium sites tend to aggregate after HER and OER; however, the metallic features remain, as confirmed by the crystal lattice in HRTEM in Figure 6f. As for Ir 4f XPS spectra (Figure 6g), the binding energies attributed to the Ir─O/P─Mo bonds undergo a positive shift for OER and a negative shift for HER, indicating the oxidation of phosphorus for forming additional Ir─O─Mo bonds after OER and the reduction of oxygen for enriching Ir─P─Mo bonds after HER. In terms of Mo 3d spectra (Figure 6h), the chemical environment is significantly changed, which is possibly due to the dissolution of molybdenum and some partial phosphorus, which can also be confirmed by P 2p XPS spectra (Figure S29, Supporting Information).

## 3 Conclusion

In conclusion, this study addresses the major challenges in acidic water electrolysis by engineering the interface between iridium active sites and a molybdenum‐based support. Through a combination of theoretical screening and rational synthesis, a catalyst system that utilizes the synergistic effects of two distinct chemical linkages, Ir‐O‐Mo and Ir‐P‐Mo, was developed. It was concluded that the covalent Ir‐P‐Mo linkage could favor HER by facilitating favorable hydrogen adsorption energy and enhanced electron transfer, while the Ir─O─Mo bond was more energetic for OER. As a result, the optimal Ir@PMoO_EtOH catalyst demonstrated superior bifunctional activity and stability in an acidic environment, evidenced by low overpotentials for both HER and OER, high mass activity, and favorable reaction kinetics. When employed in a practical two‐electrode electrolyzer, the catalyst achieved a low cell voltage of 1.501 V and outstanding long‐term durability over 250 h. This work highlights the considerable impact of tailoring metal‐support interactions at the atomic level and offers a promising design principle for creating high‐performance and cost‐effective catalysts for the scalable production of green hydrogen.

## Conflict of Interest

The authors declare no conflict of interest.

## Supporting information



Supporting Information

## Data Availability

The data that support the findings of this study are available from the corresponding author upon reasonable request.
