# Supplementary material for: Support‐Intensified Ir─P/O─Mo Cooperative Linkages for Robust Acidic Water Dissociation
Source: Adv Sci (Weinh). 2025 Dec 5;13(9):e21057. doi: 10.1002/advs.202521057 (PMC12904053; doi:10.1002/advs.202521057)
Supplement: Supplementary file 1 — Supporting Information [file ADVS-13-e21057-s001.pdf]

Supporting Information

Phosphorus-Driven Lattice Engineering on Molybdenum Interfaces for Efficient Iridium Trapping in Water Dissociation

Experimental Section

Chemicals and Materials

Polyethylene oxide-polypropylene oxide-polyethylene oxide polymer (P123) was purchased from Sigma-Aldrich. Absolute ethanol and ethylene glycol were purchased from Beijing InnoChem Science & Technology. Sodium hypophosphite monohydrate ($NaH_2PO_2 \cdot H_2O$, 98%) and Iridium trichloride hydrate ($IrCl_3$) were purchased from Macklin Biochemical Technology. Ammonium molybdate tetrahydrate ($(NH_4)_6Mo_7O_{24} \cdot 4H_2O$, 99%) was ordered from Sinopharm Chemical Reagent. Pure water used in all experiments was collected through a home-made purification system.

Synthesis of MoO_H_2O precursor

247 mg $(NH_4)_6Mo_7O_{24} \cdot 4H_2O$ powder and 200 mg P123 were dissolved in 10 mL water and 10 mL water, respectively under stirring, to obtain two transparent solutions, which were then mixed together. Subsequently, 15 mL ethylene glycol were added to the mixture, which was transferred to a 50 mL Teflon-lined stainless-steel autoclave and heated to 180 °C for 7 h. After being washed with water and ethanol, the MoO_H_2O precursor powder was collected and dried at 60 °C for 12 h. Finally, the powder was annealed at 500 °C for 1 h under nitrogen flowing, which was denoted as the MoO_H_2O precursor.

Synthesis of MoO_EtOH precursor

247 mg $(NH_4)_6Mo_7O_{24} \cdot 4H_2O$ powder and 200 mg P123 were dissolved in 10 mL water and 10 mL ethanol, respectively under stirring, to obtain two transparent solutions, which were then mixed together. Subsequently, 15 mL ethylene glycol were added to the mixture, which was transferred to a 50 mL Teflon-lined stainless-steel autoclave and heated to 180 °C for 7 h. After being washed with water and ethanol, the MoO_H_2O precursor powder was collected and dried

at 60 °C for 12 h. Finally, the powder was annealed at 500 °C for 1 h under nitrogen flowing, which was denoted as the MoO_EtOH precursor.

Synthesis of PMoO_H_2O support

60 mg as-prepared MoO_H_2O precursor and 360 mg $NaH_2PO_2 \cdot H_2O$ powder were placed on the middle stream and upstream of the quartz tube, respectively, in a tube furnace, which was then heated to 350 °C at a ramping rate of 5 °C min^{-1} under continuous nitrogen flowing and maintained at 350 °C for 2 h to obtain the PMoO_H_2O support powder.

Synthesis of PMoO_EtOH support

60 mg as-prepared MoO_EtOH precursor and 360 mg $NaH_2PO_2 \cdot H_2O$ powder were placed on the middle stream and upstream of the quartz tube, respectively, in a tube furnace, which was then heated to 350 °C at a ramping rate of 5 °C min^{-1} under continuous nitrogen flowing and maintained at 350 °C for 2 h to obtain the PMoO_EtOH support powder.

Preparation of Ir@PMoO_H_2O catalyst

5 mg as-obtained PMoO_H_2O powder was uniformly dispersed in 300 μL water under ultrasonic treatment, and then 700 μL $IrCl_3$ solution with a concentration of 10 mg mL^{-1} were added. After completely dried at 60 °C to remove water, the resultant powder was annealed at 350 °C for 1 h under continuous Ar/H_2 (95/5, v/v) flowing with a heating rate of 5 °C min^{-1} to produce the Ir@PMoO_H_2O catalyst powder.

Preparation of Ir@PMoO_EtOH catalyst

5 mg as-obtained PMoO_EtOH powder was uniformly dispersed in 300 μL water under ultrasonic treatment, and then 700 μL $IrCl_3$ solution with a concentration of 10 mg mL^{-1} were added. After completely dried at 60 °C to remove water, the resultant powder was annealed at

350 °C for 1 h under continuous Ar/H_2 (95/5, v/v) flowing with a heating rate of 5 °C min^{-1} to produce the Ir@PMoO_EtOH catalyst powder.

Material Characterizations

Inductively coupled plasma-optical emission spectrometer (ICP-OES) measurements were conducted to confirm elemental percentages of the Ir@PMoO_H_2O and Ir@PMoO_EtOH catalysts on Agilent 5800. For sample preparation, the sample was dissolved into aqua regia under continue heating for evaporation, and then the solution was cooled and diluted for tests. Powder X-ray diffraction (XRD) patterns of the MoO_H_2O and MoO_EtOH precursors, and the PMoO_H_2O and PMoO_EtOH supports were collected on a Rigaku SmartLab SE diffractometer with a copper radiation at a voltage of 40 kV and a current of 40 mA. X-ray photoelectron spectroscopy (XPS) signals of the MoO_H_2O and MoO_EtOH precursors, the PMoO_H_2O and PMoO_EtOH supports, and the Ir@PMoO_H_2O and Ir@PMoO_EtOH catalysts were identified on a Shimadzu AXIS $Supra^+$ photoelectron with an Al Kα X-ray source. Field-emission scanning electron microscopy (FE-SEM) and transmission electron microscopy (TEM) images of the MoO_H_2O and MoO_EtOH precursors, the PMoO_H_2O and PMoO_EtOH supports, and the Ir@PMoO_H_2O and Ir@PMoO_EtOH catalysts were captured on a Zeiss GeminiSEM 300, and a JEOL 2100F, respectively. Raman spectra of the MoO_H_2O and MoO_EtOH precursors, and the PMoO_H_2O and PMoO_EtOH supports were collected on a Thermo Fisher-DXR3x microscope. Fourier transform infrared spectroscopy (FT-IR) tests of the PMoO_H_2O and PMoO_EtOH supports were conducted on a Shimadzu IRTracer-100. Electron paramagnetic resonance (EPR) patterns of the MoO_H_2O and MoO_EtOH precursors, and the PMoO_H_2O and PMoO_EtOH supports were collected on a Bruker-EMXplus.

Electrochemical Measurements

Electrocatalytic properties of the Ir@PMoO_H_2O and Ir@PMoO_EtOH catalysts for acidic OER and HER were evaluated using a standard three-electrode system in 0.5M H_2SO_4 solution, in which Hg/Hg_2SO_4 as the reference electrode and graphite rod as the counter electrode, on a CHI660e workstation. Prior to catalytic measurements, several cyclic voltammetry (CV) cycles were conducted for stabilization. All linear sweep voltammetry (LSV) plots were collected with 95% iR compensation at a scan rate of 5 mV s^{-1}. Electrochemical impedance spectroscopy (EIS) plots were captured at a frequency range of 0.05-10^5 Hz. Turnover frequency (TOF) was calculated based on the equation: $TOF = \frac{|j|A}{\mathrm{m}Fn}$, where j is the current density (mA cm^{-2}); A is the electrode area (cm^2); m is the electron number consumed per molecule (4 for OER, 2 for HER); F is the Faraday constant (96485 C mol^{-1}); and n is the site number (mmol) identified by ICP-OES. Electrochemical active surface area (ECSA) was assessed by the electrochemical double-layer capacitance (C_{dl}) indicator estimated by CV technique.

Theoretical Calculation

Density functional theory (DFT) based theoretical calculations were performed in the Vienna *ab initio* simulation package (VASP) using the projector augmented plane-wave (PAW) method and the generalized gradient approximation (GGA) for exchange-correlation potential. The cut-off energy for plane wave was set to 450 eV, and the supercell structures of 4 × 4 × 1 were adopted for molecular adsorptions. The vacuum thickness is set to be 20 Å to minimize the periodic interactions. Also, the Brillouin zone integration was performed using a 4 × 4 × 1 *k*-mesh. All the structures were fully relaxed until the residual energy in iterative solution of the Kohn-Sham equation and the force reached less than 10^{-4} eV and 10^{-2} eV $Å^{-1}$, respectively. The adsorption process is described using the DFT-D3 method of Grimme with zero-damping function. Bader charge analysis was employed to quantify the charge transfer between atoms, and the visualization of the crystal structure and the charge density isosurfaces was carried out using the VESTA software.

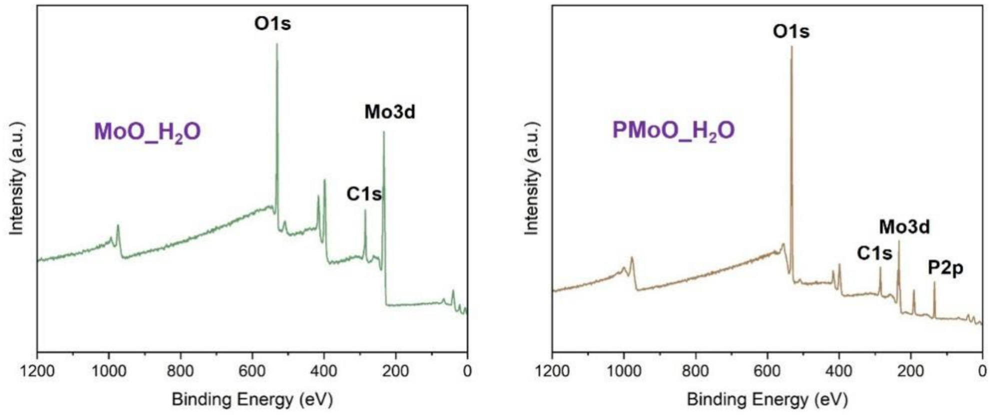

.

Figure S1. XPS survey of the MoO_H_2O precursor and the PMoO_H_2O support.

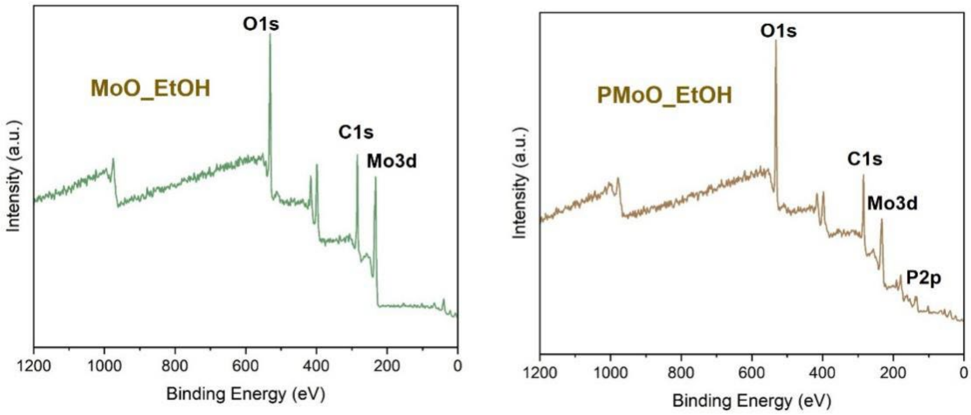

Figure S2. XPS survey of the MoO_EtOH precursor and the PMoO_EtOH support.

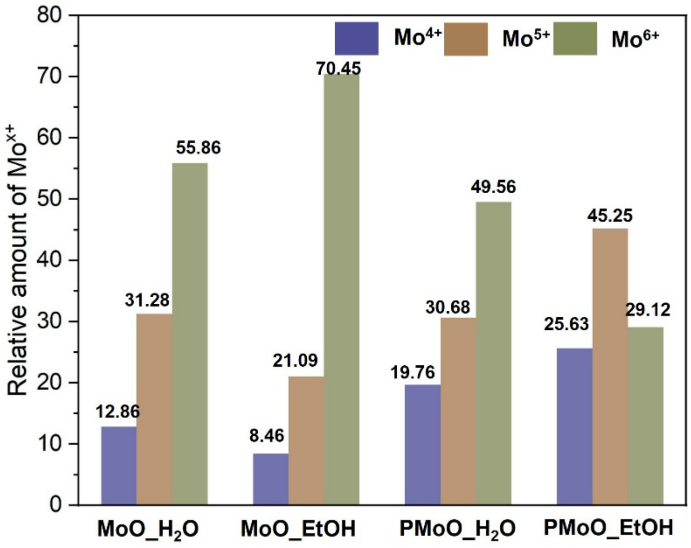

Figure S3. Percentage comparison on Mo in the precursors and supports.

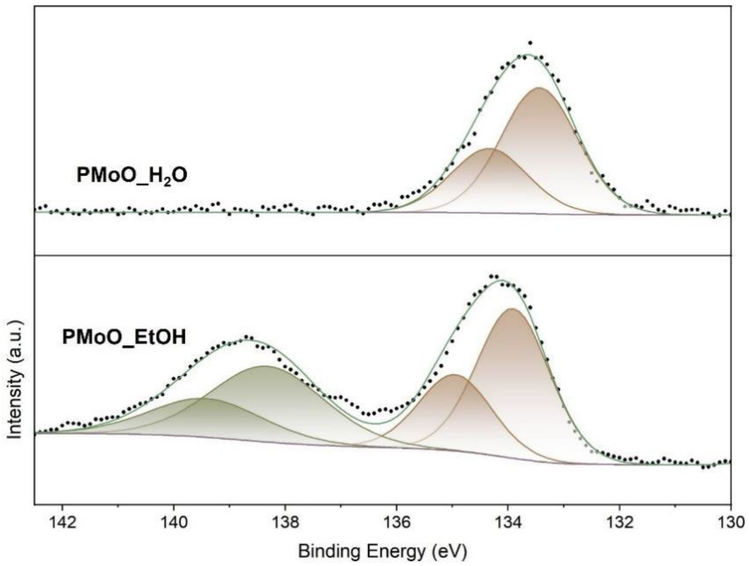

Figure S4. XPS spectra on P 2p of the PMoO_H_2O and PMoO_EtOH support.

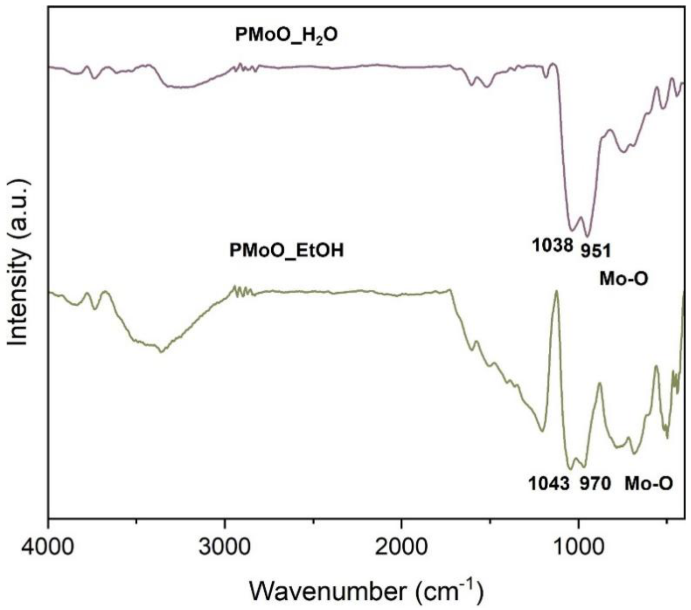

Figure S5. FT-IR spectra of the PMoO_H_2O and PMoO_EtOH support.

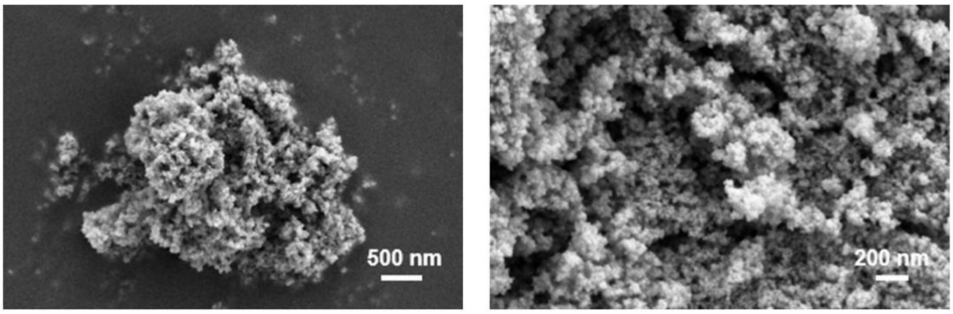

Figure S6. SEM image of the MoO_H_2O precursor and the PMoO_H_2O support.

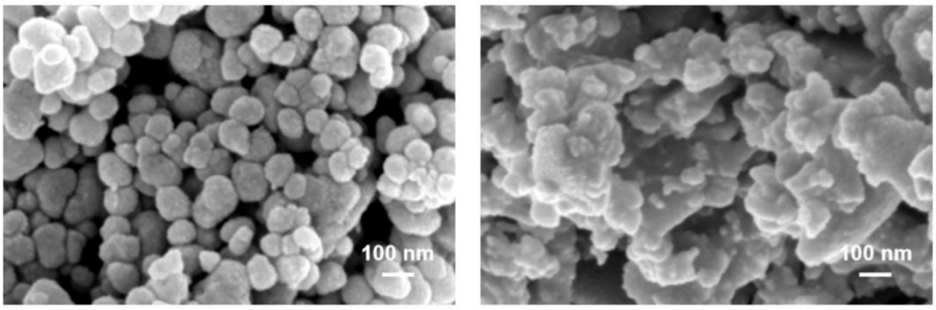

Figure S7. SEM image of the MoO_EtOH precursor and the PMoO_EtOH support.

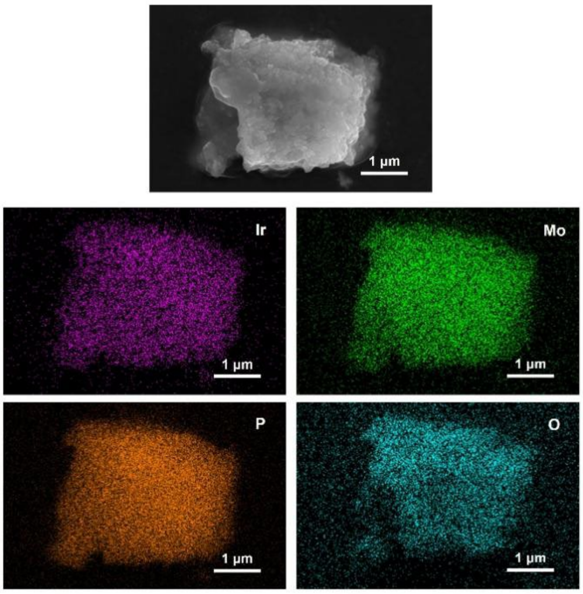

Figure S8. SEM image and element mapping patterns of the Ir@PMoO_H_2O catalyst.

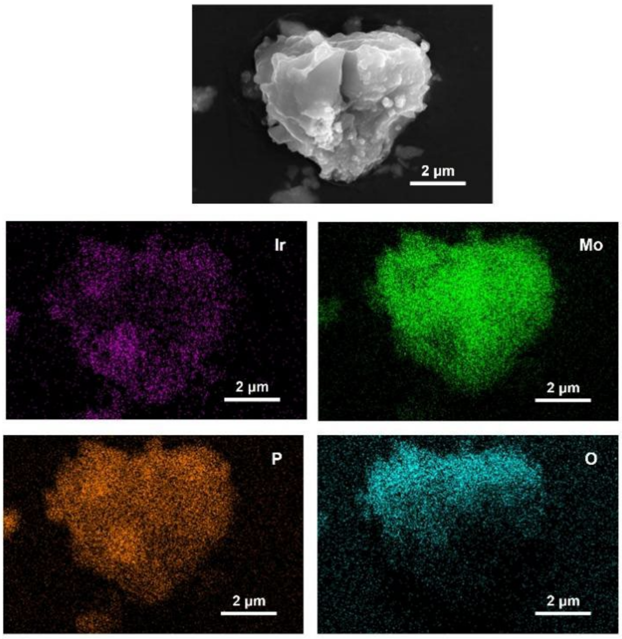

Figure S9. SEM image and element mapping patterns of the Ir@PMoO_EtOH catalyst.

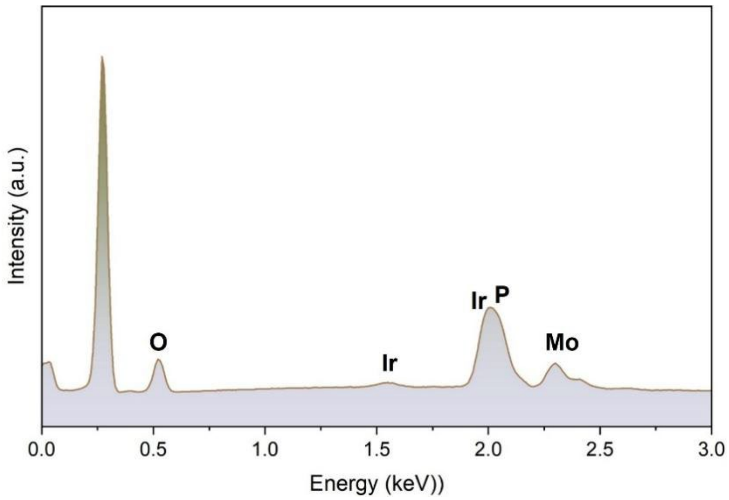

Figure S10. EDS plots of the Ir@PMoO_H_2O catalyst.

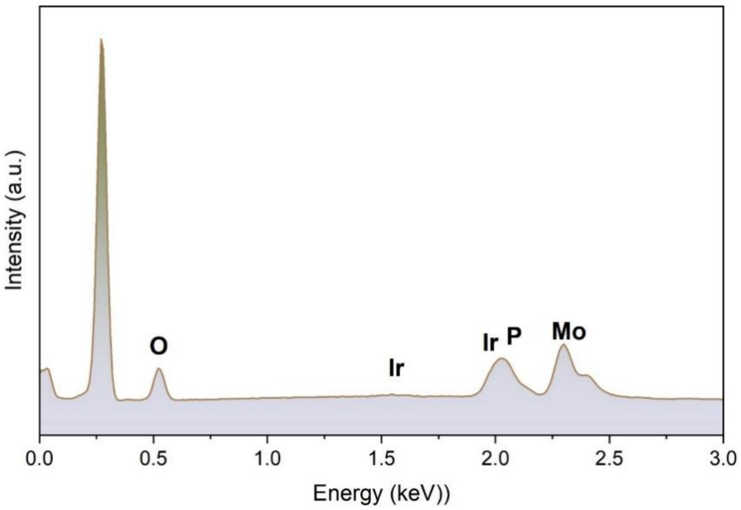

Figure S11. EDS plots of the Ir@PMoO_EtOH catalyst.

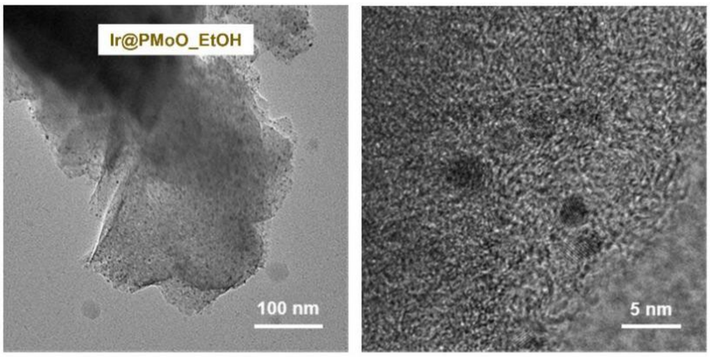

Figure S12. Low- and high-resolution TEM image of the Ir@PMoO_EtOH catalyst.

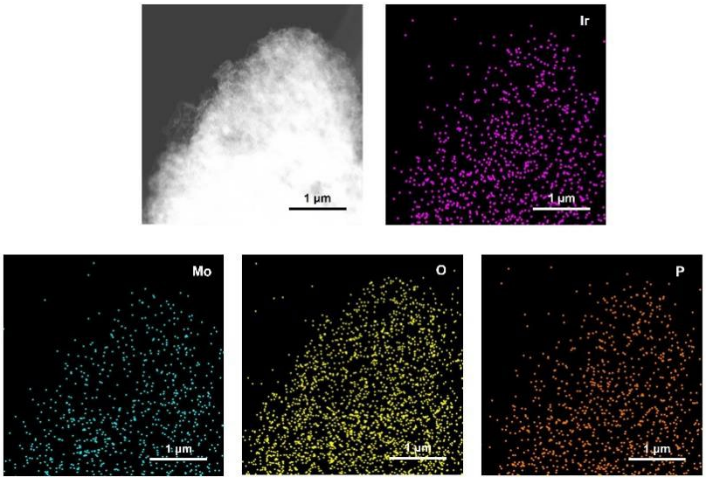

Figure S13. TEM HADDF image and element mapping pattern of the Ir@PMoO_EtOH catalyst.

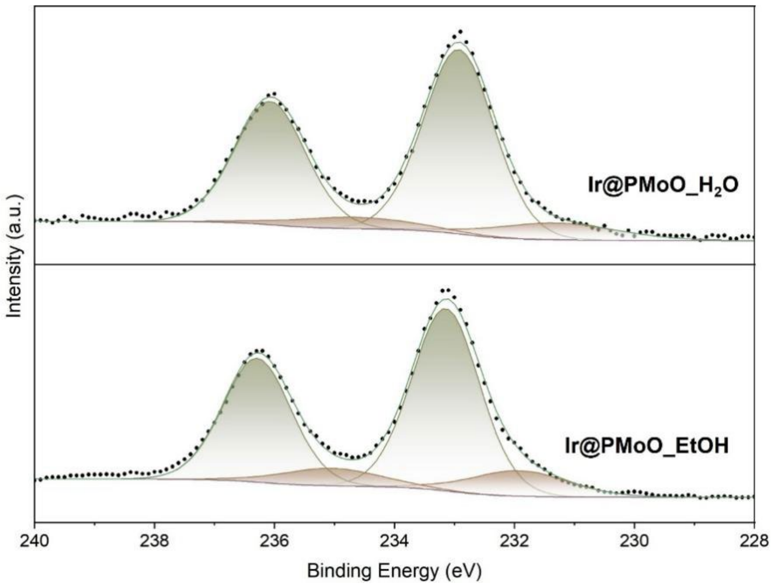

Figure S14. XPS spectra on Mo 3d of the Ir@PMoO_H_2O and Ir@PMoO_EtOH catalysts.

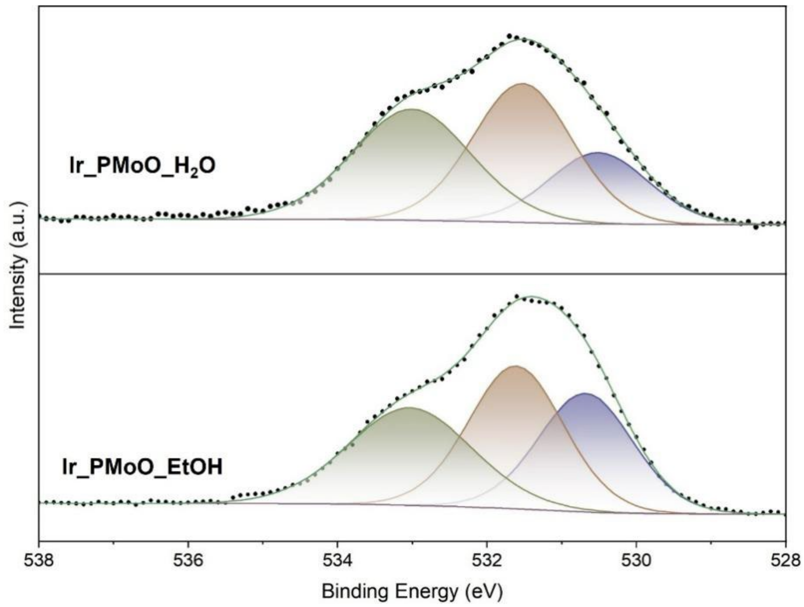

Figure S15. XPS spectra on O 2p of the Ir@PMoO_H_2O and Ir@PMoO_EtOH catalysts.

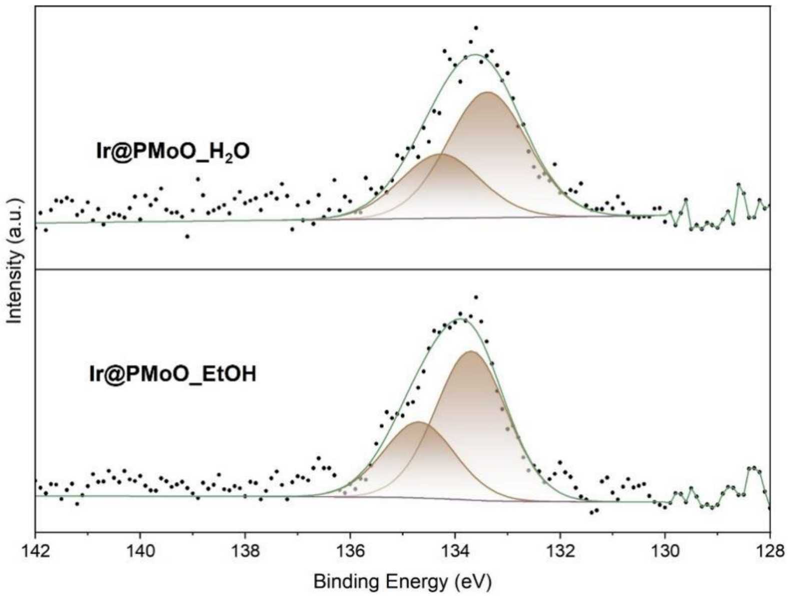

Figure S16. XPS spectra on P 2p of the Ir@PMoO_H_2O and Ir@PMoO_EtOH catalysts.

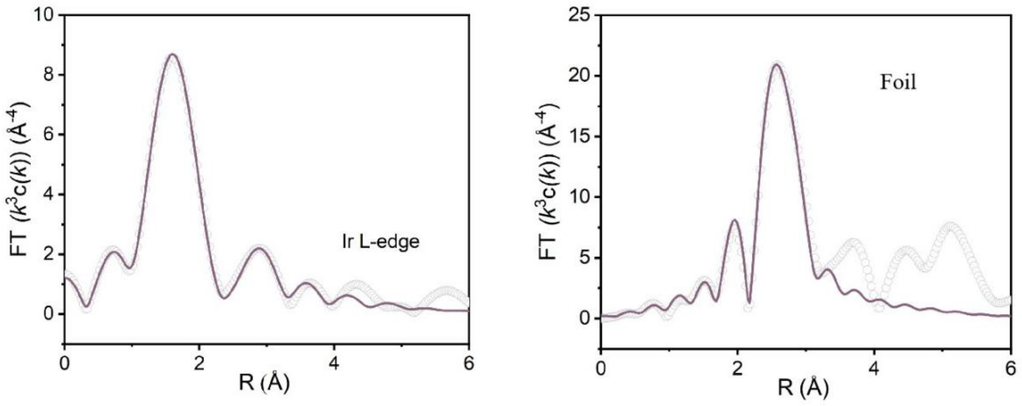

Figure S17. EXAFS refinement curves of the Ir@PMoO_EtOH catalyst and Ir foil.

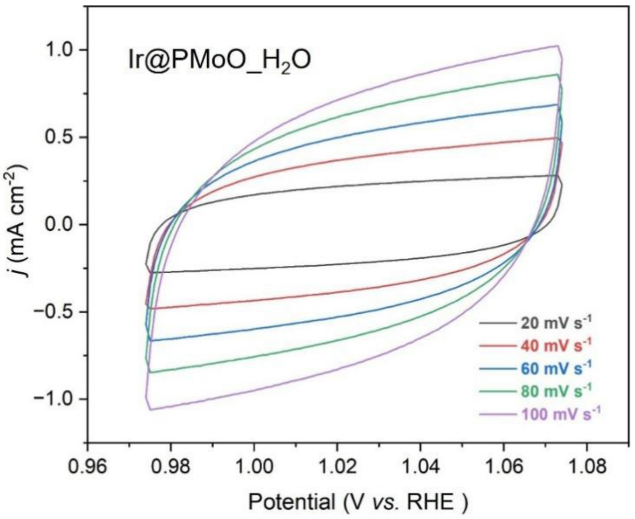

Figure S18. CV curves of the Ir@PMoO_H_2O catalyst at different scan rates.

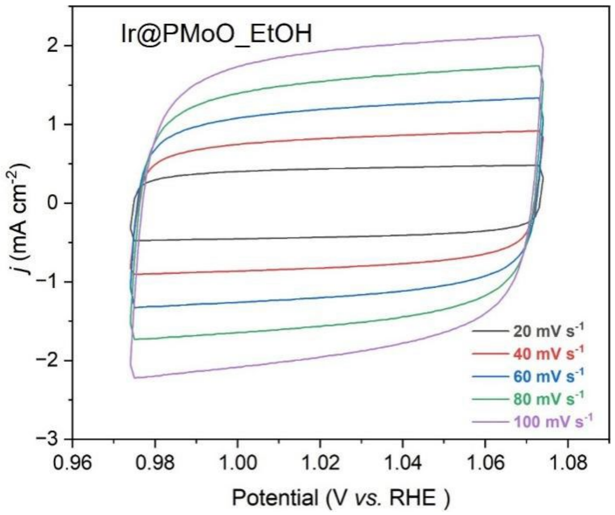

Figure S19. CV curves of the Ir@PMoO_EtOH catalyst at different scan rates.

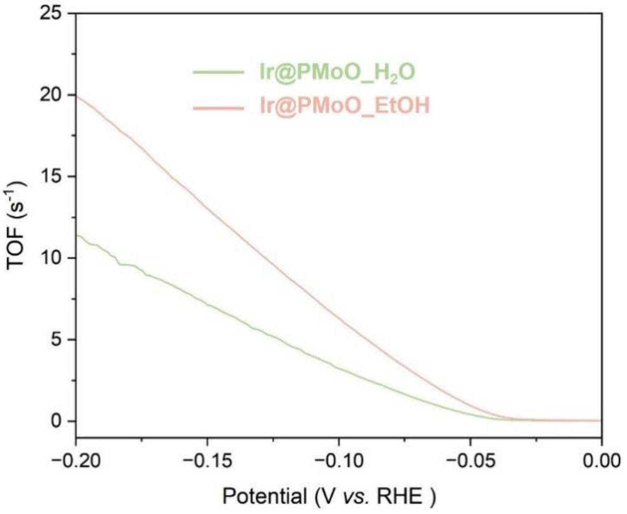

Figure S20. HER TOF plots of the Ir@PMoO_H_2O and Ir@PMoO_EtOH catalysts.

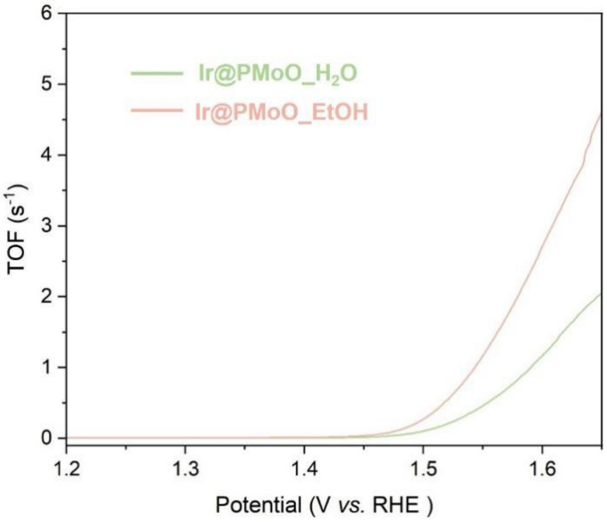

Figure S21. OER TOF plots of the Ir@PMoO_H_2O and Ir@PMoO_EtOH catalysts.

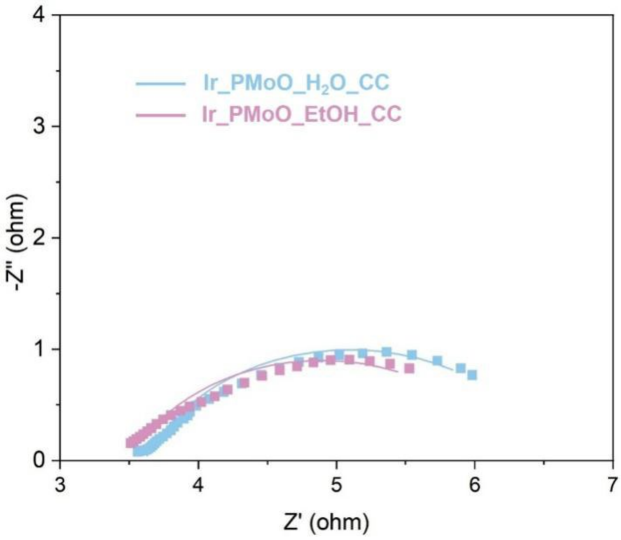

Figure S22. EIS plots of the Ir@PMoO_H_2O and Ir@PMoO_EtOH catalysts at a HER potential of -0.05 V.

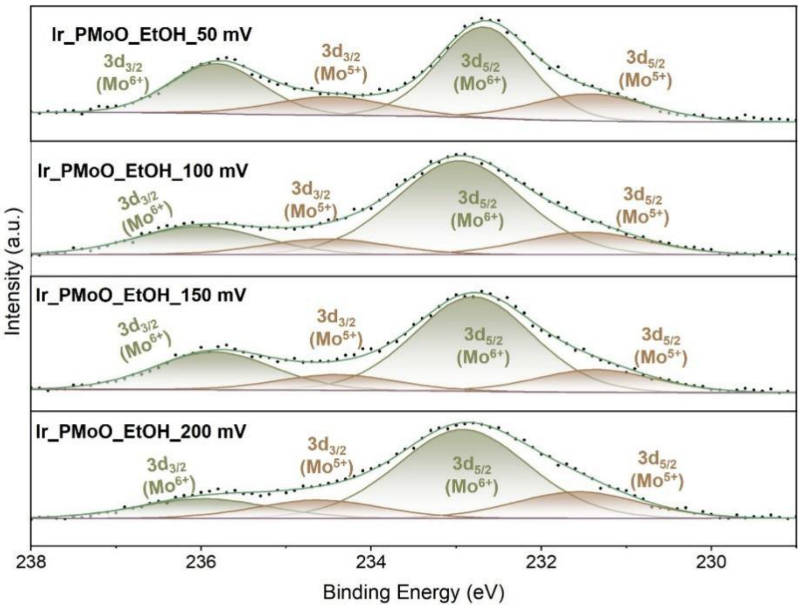

Figure S23. XPS spectra on Mo 3d of the Ir@PMoO_EtOH catalysts at different HER overpotentials.

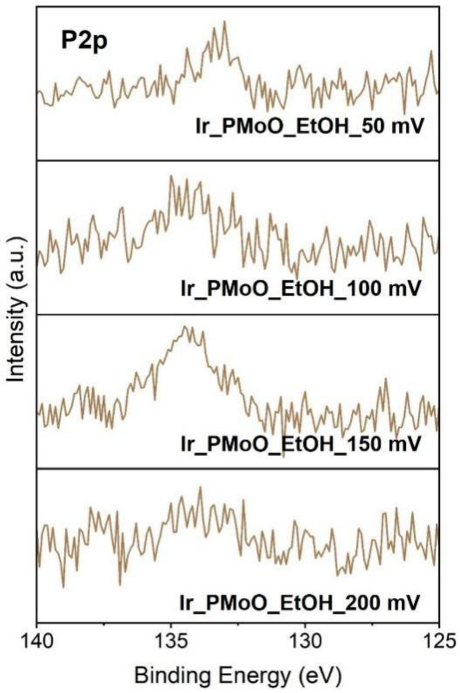

Figure S24. XPS spectra on P 2p of the Ir@PMoO_EtOH catalysts at different HER overpotentials.

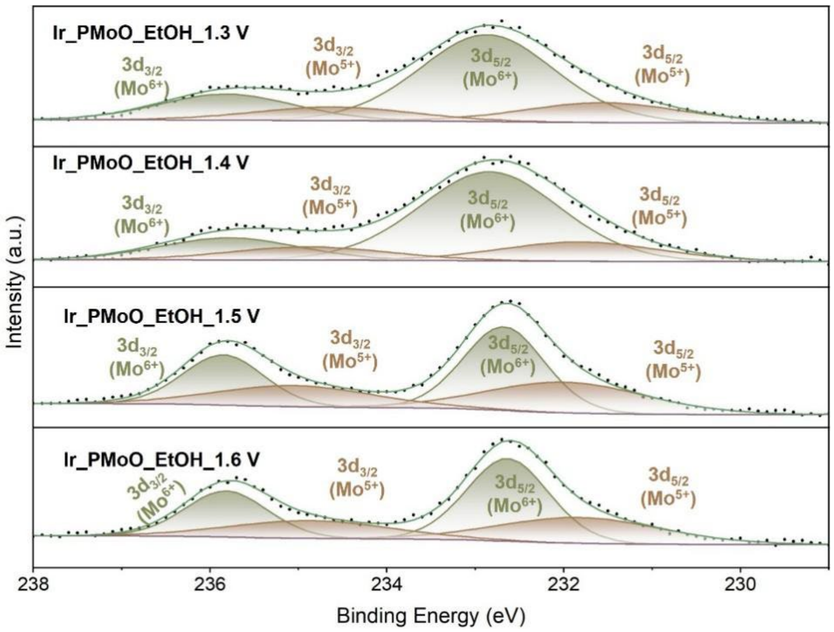

Figure S25. XPS spectra on Mo 3d of the Ir@PMoO_EtOH catalysts at different OER overpotentials.

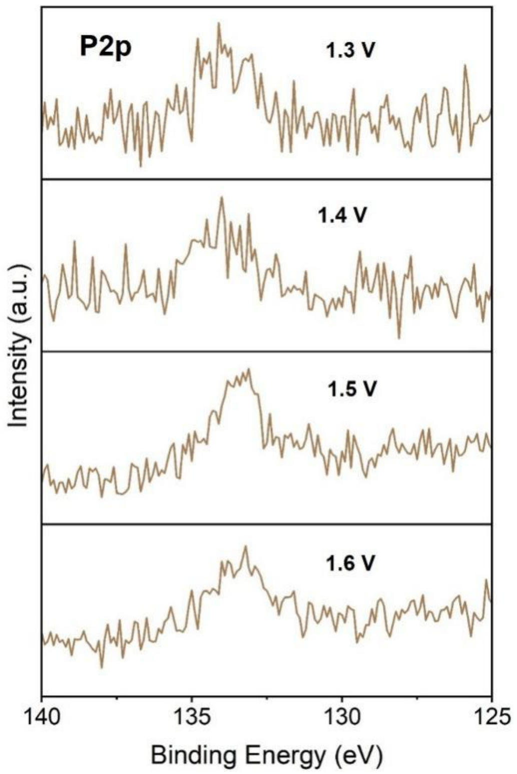

Figure S26. XPS spectra on P 2p of the Ir@PMoO_EtOH catalysts at different OER overpotentials.

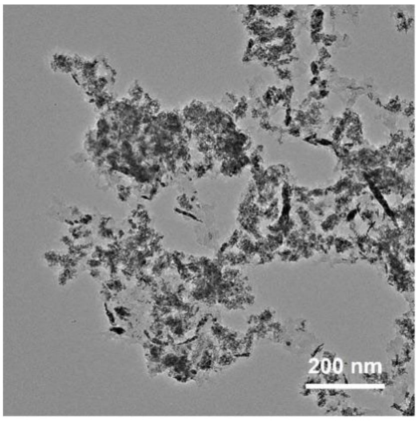

Figure S27. TEM image of the Ir@PMoO_EtOH catalyst after HER cycles.

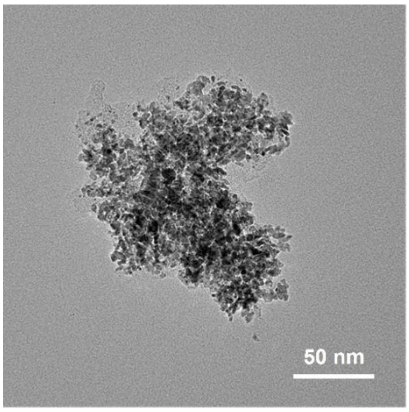

Figure S28. TEM image of the Ir@PMoO_EtOH catalyst after OER cycles.

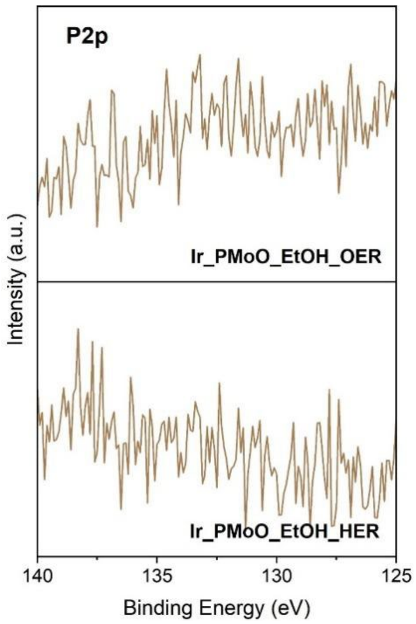

Figure S29. XPS spectra on P 2p of the Ir@PMoO_EtOH catalyst after OER cycles.

Table S1. ICP results of the Ir@PMoO_H_2O and Ir@PMoO_EtOH catalysts

**Catalysts**	**Ir (Wt%)**	**Mo (Wt%)**	**Ir/Mo (mol)**
Ir@PMoO_H_2O	5.76	12.42	1:4.32
Ir@PMoO_EtOH	3.92	8.41	1:4.30

Table S2. EXAFS Refined results of the Ir@PMoO_EtOH catalyst and Ir foil

Materials	S_0^2	shell	CN	R(Å)	σ^2 (10^{-3} Å^2)	ΔE_0	R factor
Ir foil	0.90	Ir-Ir	12	2.71±0.01	3.9±0.2	9.38±1.12	0.0157
Ir@PMoO_EtOH	0.90	Ir-O-Mo	6.2	1.93±0.01	1.9±0.3	-0.17±0.01	0.0021
		Ir-P-Mo	2.1	2.54±0.01	7.4±1.2		
		Ir-Ir	3.7	2.62±0.01	1.3±0.1		

Note: *CN*: coordination numbers; R: bond distance; σ^2: Debye-Waller factors; ΔE_0: the inner potential correction.

Table S3. Comparison of HER performance with other reported catalysts in acidic solution.

Catalysts	Electrolytes	η_{10} (mV)	Tafel (mV dec^{-1})	Ref
Ir@PMoO_EtOH@CC	0.5 M H_2SO_4.	33	37	This work
Pt-CoMoS_2/C	0.5 M H_2SO_4	118	68	[14]
$Ni_{90}Mo_{10}$/CC	0.5 M H_2SO_4	57	79	[15]
Ir-WO_3	0.5 M H_2SO_4	36	72	[16]
MoN/NC	0.5 M H_2SO_4	93	44.5	[17]
Cu@MoS_2	0.5 M H_2SO_4	160	86	[18]
Ru-MoO_{3-x}/Mo_2AlB_2	0.5 M H_2SO_4	38	57.1	[19]
Gd-MoP@C	0.5 M H_2SO_4	134	57.3	[20]
Pt@N-MoO_2	0.5 M H_2SO_4	36	43	[21]
MoP-Ru_2P/NPC	0.5 M H_2SO_4	82	65	[22]
Zn-MoC/Mo_2C	0.5 M H_2SO_4	179	66.5	[23]
FeMo@CoNi-OH/Ni_3S_2	0.5 M H_2SO_4	176	89.3	[24]
NiMo@C-CoP	0.5 M H_2SO_4	106	97	[25]
$Mo_{2.8}$-Ru@CNT-2.8	0.5 M H_2SO_4	34	48	[26]
MoS_2	0.5 M H_2SO_4	144	80.8	[27]
Ir-*g*-CN	0.5 M H_2SO_4	42	47.6	[28]
RuSAs@MoSe_2-MXene	0.5 M H_2SO_4	49	50.9	[29]
MoS_{2-x}-NbS_x	0.5 M H_2SO_4	159	53	[30]

Table S4. Comparison of OER performance with other reported catalysts in acidic solution.

**Catalysts**	**Electrolytes**	**η_{10} (mV)**	**Tafel ($mV\ dec^{-1}$)**	**Ref**
Ir_PMoO_EtOH	0.5 M H_2SO_4	249	63.0	This work
$Re_{0.1}$-IrO_2	0.5 M H_2SO_4	255	65.6	[1]
CoFeNiMoWTe	0.5 M H_2SO_4	373	66.8	[2]
Ir-rEGO	0.5 M H_2SO_4	261	70.9	[3]
Ir-MnO_2	0.5 M H_2SO_4	280	87.6	[4]
Ir-W	0.5 M H_2SO_4	364	87	[5]
$Mo_xCo_{3-x}O_4$	0.1 M $HClO_4$	420	102.5	[6]
Mo-Co_3O_4	0.5 M H_2SO_4	348	84.3	[7]
IrBa-Co_3O_4	0.5 M H_2SO_4	249	65.3	[8]
IrO_x/TiO_2	0.5 M H_2SO_4	303	90.3	[9]
Mo-Co_3O_4	0.5 M H_2SO_4	310	82.3	[10]
Ir@N-G-600	0.5 M H_2SO_4	315	74	[11]
IrCo NRAs	0.5 M H_2SO_4	297	68.1	[12]
Ir-PdO	0.5 M H_2SO_4	277	74	[13]

Table S5. Comparison of performances with other reported electrocatalysts in acidic solution.

Two-electrode Cells	Electrolytes	Voltage (V)	Ref
Ir@PMoO_EtOH_CC‖Ir@PMoO_EtOH_CC	0.5 M H_2SO_4	1.501	This work
$IrNiO_x/WO_3$‖$IrNiO_x/WO_3$	0.5 M H_2SO_4	1.539	[31]
$RuIrO_x$@NHC‖$RuIrO_x$@NHC	0.5 M H_2SO_4	1.53	[32]
Ir-WO_3‖Ir-WO_3	0.5 M H_2SO4	1.56	[16]
Au@$Au_{0.43}Ir_{0.57}$‖Au@$Au_{0.43}Ir_{0.57}$	0.5 M H_2SO_4	1.52	[33]
CoFeNiMoWTe‖Pt/C	0.5 M H_2SO_4	1.63	[2]
Ir-rEGO‖Ir-rEGO	0.5 M H_2SO_4	1.51	[3]
$IrIn_2$/C‖$IrIn_2$/C	0.5 M H_2SO_4	1.51	[34]
Ir-W‖Ir-W	0.5 M H_2SO_4	1.54	[5]
Ru@$MoO(S)_3$‖Ru@$MoO(S)_3$	0.5 M H_2SO_4	1.522	[35]
Ir/MoS_2 NFs‖Ir/MoS_2 NFs	0.5 M H_2SO_4	1.55	[36]
IrBa-Co_3O_4‖Pt/C	0.5 M H_2SO_4	1.57	[8]
IrCo@NCNT/PC‖IrCo@NCNT/PC	0.5 M H_2SO_4	1.51	[37]
MoS_{2-x}-NbS_x‖MoS_{2-x}-NbS_x	0.5 M H_2SO_4	1.659	[30]
IrTe NTs‖IrTe NTs	0.5 M H_2SO_4	1.53	[38]

Reference

[1] W. Huo, X. Zhou, Y. Jin, C. Xie, S. Yang, J. Qian, D. Cai, Y. Ge, Y. Qu, H. Nie, Z. Yang, *Small* **2023**, *19*, 2207847.

[2] S. Jo, M. C. Kim, K. B. Lee, H. Choi, L. Zhang, J. I. Sohn, *Adv. Energy Mater.* **2023**, *13*, 2301420.

[3] X. X. Li, J. Cao, J. Chen, Y. Zhu, H. Xia, Z. Xu, C. Gu, J. Xie, M. Jones, C. Lyu, J. Corbin, X. X. Li, W. Hu, *Adv. Funct. Mater.* **2024**, *34*, 2313530.

[4] Y. Sun, J. Chen, L. Liu, H. Chi, H. Han, *Chinese J. Catal.* **2025**, *69*, 99.

[5] W. Shao, Y. Zhang, R. Yan, T. Ma, S. Li, *J. Mater. Chem. A* **2025**, 5821.

[6] L. Sun, M. Feng, Y. Peng, X. Zhao, Y. Shao, X. Yue, S. Huang, *J. Mater. Chem. A* **2024**, *12*, 8796.

[7] R. Fang, X. Wan, J. Chen, Y. Hou, B. Hua, *Green Chem.* **2025**, *27*, 9243.

[8] M. Huo, Q. Li, Y. Liang, W. Liu, H. Wang, K. Qin, X. Sun, Y. Ma, Z. Xing, J. Chang, *Chem. Eng. J.* **2025**, *519*, 165555.

[9] Q. Chu, Y. Niu, H. Tao, H. Liu, Q. Li, C. Lian, J. Li, *ACS Catal.* **2025**, *15*, 1942.

[10] Z. Liu, W. Shen, X. Liang, J. Liang, K. Xu, X. Ge, J. Lu, H. Liu, J. Xiao, L. Wang, J. Gao, J. Liu, *ACS Catal.* **2025**, *15*, 8943.

[11] L. Yi, B. Feng, N. Chen, W. Li, J. Li, C. Fang, Y. Yao, W. Hu, *Chem. Eng. J.* **2021**, *415*, 129034.

[12] Y. Zhang, G. Zhang, M. Zhang, X. Zhu, P. Shi, S. Wang, A. L. Wang, *Chem. Eng. J.* **2022**, *433*, 133577.

[13] Y. Wang, J. Jiang, Z. Shi, H. Wu, J. Yang, P. Wang, S. Hou, M. Xiao, J. Ge, C. Liu, W. Xing, *Nano Res.* **2024**, *17*, 2492.

[14] L. A. Zavala, K. Kumar, V. Martin, F. Maillard, F. Maugé, X. Portier, L. Oliviero, L. Dubau, *ACS Catal.* **2023**, *13*, 1221.

[15] J. Li, W. Hu, L. Sun, L. Zhang, Q. Zhang, X. Ren, Y. Li, *Small* **2024**, *20*, 2403364.

[16] P. Li, X. Duan, Y. Kuang, X. Sun, *Small* **2021**, *17*, 2102078.

[17] C. Huang, X. L. Zhang, J. Tang, D. Li, Q. D. Ruan, L. L. Liu, F. Y. Xiong, B. Wang, Y. Xu, S. H. Cui, Y. Luo, Q. W. Li, P. K. Chu, *Rare Met.* **2023**, *42*, 1446.

[18] Z. Li, X. Yan, D. He, W. Hu, S. Younan, Z. Ke, M. Patrick, X. Xiao, J. Huang, H. Wu, X. Pan, J. Gu, *ACS Catal.* **2022**, *12*, 7687.

[19] Y. Yang, D. Pang, C. Wang, Z. Fu, N. Liu, J. Liu, H. Wu, B. Jia, Z. Guo, X. Fan, J. Zheng, *Angew. Chem. Int. Ed.* **2025**, e202504084.

[20] J. Li, J. Zhao, Y. Zhang, Y. Liu, M. Li, R. Ge, W. Li, B. Liu, *Adv. Sci.* **2025**, *12*, 2417583.

[21] L. Wang, C. Zhang, Z. Cao, G. Zeng, J. Liu, S. Ye, *Adv. Funct. Mater.* **2024**, *34*, 2406670.

[22] Y. Gao, Z. Chen, Y. Zhao, W. Yu, X. Jiang, M. He, Z. Li, T. Ma, Z. Wu, L. Wang, *Appl. Catal. B Environ.* **2022**, *303*, 120879.

[23] C. Yang, R. Zhao, H. Xiang, J. Wu, W. Zhong, X. Li, Q. Zhang, *Nano Energy* **2022**, *98*, 107232.

[24] W. Fang, Y. Wu, S. Xin, Y. Hu, J. Dang, M. Li, B. Chen, H. Zhao, Z. Li, *Chem. Eng. J.* **2023**, *468*, 143605.

[25] H. Luo, X. Zhang, H. Zhu, K. Zhang, F. Yang, K. Xu, S. Yu, D. Guo, *J. Mater. Sci. Technol.* **2023**, *166*, 164.

[26] W. Xu, D. Zhang, T. Wang, J. Lai, L. Wang, *Appl. Catal. B Environ.* **2025**, *361*, 124626.

[27] M. K. Kim, B. Lamichhane, B. Song, S. Kwon, B. Wang, S. Kattel, J. H. Lee, H. M. Jeong, *Appl. Catal. B Environ. Energy* **2024**, *352*, 124037.

[28] Z. Yu, Y. Li, A. Torres-Pinto, A. P. LaGrow, V. M. Diaconescu, L. Simonelli, M. J. Sampaio, O. Bondarchuk, I. Amorim, A. Araujo, A. M. T. Silva, C. G. Silva, J. L. Faria, L. Liu, *Appl. Catal. B Environ.* **2022**, *310*, 12318.

[29] T. Ma, P. Wang, H. J. Niu, Z. Che, G. Li, W. Zhou, *Carbon* **2024**, *218*, 118758.

[30] M. Naseem, M. Tahir, J. Dai, L. Qu, F. U. Nisa, W. Ahmad, I. Shahbaz, Z. Ma, A. U. Khan, L. He, *Small* **2025**, *21*, 2501464.

[31] H. Zhang, P. Song, X. Mei, D. Zhang, C. Liu, M. Chu, T. Zhang, C. Han, W. Xu, *ACS Catal.* **2025**, *15*, 12395.

[32] Z. Lu, H. Yang, G. Qi, Q. Liu, L. Feng, H. Zhang, J. Luo, X. Liu, *Small* **2024**, *20*, 2308841.

[33] H. Wang, Z. N. Chen, Y. Wang, D. Wu, M. Cao, F. Sun, R. Cao, *Natl. Sci. Rev.* **2024**, *11*, nwae056.

[34] C. He, C. Ma, J. Xia, H. Zhang, S. Han, Y. Tian, A. L. Wang, X. Meng, W. Cao, Q. Lu, *Adv. Funct. Mater.* **2024**, *34*, 2311683.

[35] D. Chen, R. Yu, D. Wu, H. Zhao, P. Wang, J. Zhu, P. Ji, Z. Pu, L. Chen, J. Yu, S. Mu, *Nano Energy* **2022**, *100*, 107445.

[36] C. Wang, L. Yu, F. Yang, L. Feng, *J. Energy Chem.* **2023**, *87*, 144.

[37] D. Zhao, Y. Zhu, Q. Wu, W. Zhou, J. Dan, H. Zhu, W. Lei, L. J. Ma, L. Li, *Chem. Eng. J.* **2022**, *430*, 132825.

[38] Z. Wang, P. Wang, H. Zhang, W. Tian, Y. Xu, X. Li, L. Wang, H. Wang, *J. Mater. Chem. A* **2021**, *9*, 18576.
